# Effects of metformin administration on endocrine-metabolic parameters, visceral adiposity and cardiovascular risk factors in children with obesity and risk markers for metabolic syndrome: A pilot study

**DOI:** 10.1371/journal.pone.0226303

**Published:** 2019-12-10

**Authors:** Judit Bassols, José-María Martínez-Calcerrada, Inés Osiniri, Ferran Díaz-Roldán, Silvia Xargay-Torrent, Berta Mas-Parés, Estefanía Dorado-Ceballos, Anna Prats-Puig, Gemma Carreras-Badosa, Francis de Zegher, Lourdes Ibáñez, Abel López-Bermejo

**Affiliations:** 1 Maternal-Fetal Metabolic Group, [Girona Biomedical Research Institute] IDIBGI, Salt, Spain; 2 Clinical Laboratory, Salut Empordà Foundation, Figueres, Spain; 3 Pediatrics, Dr. Trueta University Hospital, Girona, Spain; 4 Pediatric Endocrinology Group, [Girona Biomedical Research Institute] IDIBGI, Salt, Spain; 5 Department of Physical Therapy, EUSES University School, University of Girona, Girona, Spain; 6 Department of Development & Regeneration, University of Leuven, Leuven, Belgium; 7 Endocrinology, Pediatric Research Institute, Sant Joan de Déu Children’s Hospital, Barcelona, Spain; 8 [Spanish Biomedical Research Centre in Diabetes and Associated Metabolic Disorders] CIBERDEM, ISCIII, Madrid, Spain; Rush University, UNITED STATES

## Abstract

**Background:**

Metformin treatment (1000–2000 mg/day) over 6 months in pubertal children and/or adolescents with obesity and hyperinsulinism is associated with a reduction in body mass index (BMI) and the insulin resistance index (HOMA-IR). We aimed to ascertain if long-term treatment (24 months) with lower doses of metformin (850 mg/day) normalizes the endocrine-metabolic abnormalities, improves body composition, and reduces the carotid intima-media thickness (cIMT) in pre-puberal and early pubertal children with obesity.

**Methods:**

A pilot double-blind, placebo-controlled trial was conducted on 18 pre-puberal and early pubertal (Tanner stage I-II) children with obesity and risk markers for metabolic syndrome. Patients were randomly assigned (1:1) to receive metformin (850 mg/day) or placebo for 24 months. Clinical, biochemical (insulin, lipids, leptin, and high-sensitivity C-reactive protein [hsCRP]), and imaging (body composition [dual-energy X-ray absorptiometry and magnetic resonance imaging]) parameters as well as cIMT (ultrasonography) were assessed at baseline and at 6, 12, and 24 months.

**Results:**

The 12-month treatment tend to cause a reduction in weight standard deviation scores (SDS), BMI-SDS, leptin, leptin–to–high-molecular-weight (HMW) adiponectin ratio, hsCRP, cIMT, fat mass, and liver fat in metformin-treated children compared with placebo. The effect of metformin on the reduction of BMI-SDS, leptin, leptin-to-HMW adiponectin ratio, hsCRP, and liver fat seemed to be maintained after completing the 24 months of treatment. No changes in insulin sensitivity (HOMA-IR) or adverse effects were detected.

**Conclusion:**

In this pilot study, metformin treatment in pre-puberal and early pubertal children with obesity seemed to improve body composition and inflammation markers. Our data encourage the development of future fully powered trials using 850 mg/day metformin in young children, highlighting its excellent tolerance and potential long-term benefits.

## Introduction

Obesity is an important public health problem, owing to the exponential increase in its prevalence in recent years [1]; indeed, the prevalence of pediatric obesity has tripled in one decade in Spain [2]. Obesity is an independent risk factor for metabolic (dyslipidemia, type 2 diabetes, metabolic syndrome) and cardiovascular diseases (hypertension, atherosclerosis) [3].

The mainstay of prevention and treatment of obesity in children is based on lifestyle modification including diet and physical activity; however, long-term compliance to these remains very poor. While some of these programs have shown to be effective in improving anthropometric parameters, there is conflicting evidence about their effectiveness in improving the body composition and reversing the insulin resistance and metabolic derangements [4, 5]. Therefore, different pharmacological therapies have been used to treat childhood obesity and its complications [6].

Metformin is a biguanide widely used to treat type 2 diabetic patients [7]. It has been successfully used to reduce weight gain, hyperinsulinemia, and dyslipidemia in adults, and in patients with prediabetes, to prevent the progression from glucose intolerance to type 2 diabetes [8, 9]. These results have prompted the use of metformin in adolescents in the early phases of development of diabetes and metabolic syndrome, mainly in those patients with obesity and hyperinsulinemia, in order to reduce the risk for metabolic and cardiovascular diseases [10]. In pre-puberal girls with a history of low birth weight (LBW, who are at increased risk for metabolic syndrome in adulthood), increased visceral adiposity, and risk markers for metabolic syndrome, treatment with metformin before and throughout puberty decreases the gains in total, visceral, and hepatic adiposity and prevents the deterioration of endocrine-metabolic parameters [11]. In these girls, metformin treatment was followed by a delayed progression of puberty, a more normal menarcheal age, and a taller adult height [12, 13].

Recent reviews including a total of 15 randomized controlled trials (RCTs) with metformin treatment (1000–2000 mg/day) over 6 months in children and/or adolescents with obesity and hyperinsulinemia reported that more than 50% of the studies showed a greater reduction in body mass index (BMI) with metformin versus controls (average reduction of -1.3 kg/m^2^) and about 25% of the studies showed a significant reduction in HOMA-IR in the metformin versus control group (average reduction of -0.6) [6, 14]. Adverse events were reported to have occurred in all metformin trials, with gastrointestinal side effects the most commonly reported. A similar RCT in pre-puberal and pubertal children with obesity showed that metformin (1000 mg/day for 6 months) decreased the BMI z score and improved inflammatory and cardiovascular-related obesity parameters in pre-puberal children but not in pubertal children [15].

Puberty, together with prenatal and early postnatal life, are highly dynamic periods characterized by a plasticity of the body to adapt to the increased metabolic and growing demands of these phases of life [16]. Our previous studies in pre-puberal girls with LBW without obesity but with an increase in visceral adiposity and cardiometabolic risk markers showed that pre-puberal metformin treatment not only prevents the worsening of the metabolic profile in these girls but has persistent effects beyond this period of life, once the pharmacological treatment has been discontinued [17]. Although a post-pubertal onset of metformin treatment also improves the metabolic profile in these girls, metformin’s normalizing effects are reversed as soon as the drug is discontinued [18]. It has therefore been proposed that puberty offers a window of opportunity for reprogramming, the metabolic abnormalities of these patients in order to delay a potential development of metabolic syndrome.

The above-mentioned data obtained in patients with obesity provide enough evidence to sustain beneficial effects of metformin in pediatric populations. However, these RCTs evaluated the effect of 1000–2000 mg/day metformin over 6 months in pubertal children and/or adolescents with obesity, focusing on anthropometric and metabolic parameters. Only six of these RCTs had an intervention of more than 6 months, and they found no further improvement in BMI of metformin treated groups, though their BMI was lower than the controls [14].

Here, we performed a pilot study to ascertain whether a lower dose of metformin (850 mg/day) for 24 months normalizes the endocrine-metabolic abnormalities, improves body composition (specifically, decreases the excess of visceral and liver fat), and reduces carotid intima-media thickness in pre-puberal and early pubertal children with obesity.

## Materials and methods

### Participants, study design, and ethics

This was a randomized, double-blind, placebo-controlled clinical trial with parallel groups (one treated with metformin and the other one with placebo as a control) conducted at Hospital Dr. Josep Trueta of Girona, Spain, between 21st December 2011 and 31st December 2017. The study was registered as EC10-252 (EudraCT number: 2010-024414-61; clinicaltrialsregister.eu) and was conducted without support from pharmaceutical industry and after approval by the Institutional Review Board of Hospital Dr Josep Trueta and the Spanish Agency of Medicines and Health Products–AEMPS (Ministry of Health). Informed written consent was obtained from the parents.

Participants were pre-puberal and early pubertal Caucasian children aged 6 to 13 years, of both sexes, with obesity, unfavorable body composition, and risk markers for metabolic syndrome at inclusion, as described below. The patients were recruited by phone or letter, according to computerized records of the health area, and a first appointment was given for a prescreening visit in those families interested in the study. At the prescreening visit, a trained nurse checked whether the children fulfilled the initial inclusion criteria, including current auxological data (BMI) and auxological data at birth (weight, length, and gestational age) in order to sign the informed written consent. Patients fulfilling the initial inclusion criteria underwent imaging and blood tests and were appointed for the screening visit to verify the compliance of all the inclusion criteria and absence of all the exclusion criteria.

The inclusion criteria were (all had to be fulfilled): (1) age between 6 and 13 years; (2) pre-puberal or early pubertal (Tanner stage I-II) [19, 20]; (3) BMI between 2 SD (97th centile) and 4 SD, for age and sex; (4) fasting insulin levels >6 mIU/L; (5) visceral-to-subcutaneous fat ratio (magnetic resonance imaging [MRI]) >90th centile, based on a reference of healthy children without obesity [21]; and (6) birth weight above –1.5 SD and below +1.5 SD for gestational age, to avoid the influence of birth weight deviations on metabolic and cardiovascular risk markers. The BMI of the patient had to be stable (along the same percentile) for the past 3 months prior to inclusion in the trial.

The exclusion criteria were (any of the following): (1) drug or alcohol abuse during gestation; (2) genetic syndromes; and (3) hypothalamic obesity (previous hypothalamic damage, diagnosis of Prader-Willi syndrome, or side effects of psychotropic drugs). At the time of inclusion: (4) abnormalities in thyroid, liver, or renal function or in serum electrolytes; (5) known skin allergies; (6) glucose intolerance or type 2 diabetes; (7) chronic illnesses other than obesity; (8) treatment with corticosteroids, sexual hormones, and drugs that may alter glucose tolerance or insulin sensitivity; (9) acute infections or use of anti-inflammatory drugs or antibiotics 2 weeks prior to potential inclusion in the study; (10) medical treatment or other therapies aimed at reducing body weight (3 months prior to potential inclusion in the study).

The children were randomly assigned (1:1) to receive metformin (850 mg, metformin KERN Pharma SL) or placebo (manufactured by the same company: KERN Pharma SL) once daily, at dinner time for 24 months. Follow-up visits and additional examinations were made at baseline and at 6, 12, and 24 months on treatment. Side effects were assessed at each follow-up visit and also at 18 months by direct interview with the parents and children (usually if they were 12 years of age or older). The case report form included a list of possible clinical events in between visits affecting the gastrointestinal, cardiovascular, musculoskeletal, neurological or psychiatric systems, among others.

The randomization was performed at Hospital Dr. Josep Trueta by an independent investigator and was based on random permuted blocks and strata for age, gender, and BMI. Both treatments were dispensed in identically labeled boxes with the same appearance by the pharmacy of Dr. Hospital Josep Trueta, precluding the identification of the allocated therapy. The list of the contents and numbers of boxes was not uncovered until the end of the study. Medication compliance was calculated at the end of the study as [(pills ingested–pills returned)/pills prescribed] × 100.

The primary endpoints were fasting insulin, insulin sensitivity, visceral and liver fat, visceral-to-subcutaneous fat ratio, and carotid intima-media thickness (cIMT). We considered as positive and discriminative responses an increase of more than 30% in insulin sensitivity (estimated by the HOMA-IR method), a decrease of more than 15% in fasting insulin, a decrease of at least 10% in visceral or liver fat or in the visceral-to-subcutaneous fat ratio, and a decrease of at least 15% in cIMT. The dose of medication and the estimated effects on the primary endpoints were selected according to previous results from the group in adolescents with a history of precocious puberty and low birth weight, hyperinsulinism, subclinical hyperandrogenism and dyslipidemia, treated with metformin [12, 13].

To detect significant changes (with a potency of 80% and a significance level of *p* < 0.05) in the primary outcomes between the metformin- and the placebo-treated groups, a total of 56 patients were needed. The study was designed to include 80 patients in order to account for potential patient dropouts on follow-up (which was estimated to be up to 30%). Even though we increased the expected recruitment time from 12 months to 2 years, it became clear that only a small number of patients fulfilled the stringent inclusion and exclusion criteria; consequently, we were allowed to enroll a total of 22 children. During the first 6 months, four participants in the metformin group dropped out of the study, and the total number of assessed children decreased to a total of 18 children. As a consequence, the study was reconsidered to be a preliminary, pilot study.

### Anthropometric and clinical assessments

Clinical examination and venous blood sampling were performed in the morning and in the fasting state. Weight was measured wearing light clothes with a calibrated scale, and height was measured with a Harpenden stadiometer. Age- and sex-adjusted standard deviation scores for current BMI (BMI-SDS) were calculated using regional normative data [22]. Waist and hip circumferences were measured in the supine position at the umbilical and around the widest portion of the buttocks, respectively.

Serum endocrine-metabolic parameters were measured using automated tests, as described before [23]. Serum glucose was measured by the hexokinase method. Insulin was measured by immunochemiluminiscence (IMMULITE 2000, Diagnostic Products, Los Angeles, CA, USA). The lower detection limit was 0.4 mIU/L, and the intra- and interassay coefficients of variation (CVs) were <10%. Insulin sensitivity was estimated from fasting insulin and glucose levels using the homeostasis model assessment [HOMA-IR = (fasting insulin in mIU/L) × (fasting glucose in mM)/22.5]. Total serum triacylglycerol was measured by monitoring the reaction of glycerol-phosphate oxidase (ARCHITECT, Abbott Laboratories, Abbott Park, IL; lower detection limit of 5.0 mg/dL and intra- and inter-assay CVs <5%), and high-density lipoprotein cholesterol was quantified by a homogenous method of selective detergent with accelerator (ARCHITECT, Abbott Laboratories, Abbott Park, IL; lower detection limit of 2.5 mg/dL and intra- and interassay CVs <4%). Serum levels of high-sensitivity C-reactive protein (hsCRP) were measured using the ultrasensitive latex immunoassay CRP Vario (Sentinel Diagnostics, Abbott Diagnostics Europe, Milan, Italy; lower detection limit of 0.2 mg/L and intra- and interassay CVs <3%). High-molecular weight (HMW) adiponectin and leptin were assessed with sandwich enzyme-linked immunosorbent assay kits (HMW adiponectin: Linco Research, St. Charles, MO, USA; lower detection limit of 0.5 ng/mL and intra- and interassay CVs <4%; and leptin: Millipore, Massachusetts, USA; lower detection limit of 0.2 ng/mL and intra- and interassay CVs <10%).

### High-resolution ultrasound measurements

cIMT was measured by high-resolution ultrasonography (MyLab^™^25, Esaote, Firenze, Italy). Diastolic images were obtained using a linear 7.5- to 12-MHz transducer on the right side at the level of the distal common carotid artery, 1 cm away from its bifurcation [24]. Averages of five cIMT measurements on the far wall of the artery were used in the study. All measurements were performed by the same operator who was blinded to treatment allocation. Intrasubject CVs were less than 6%.

### DXA and MRI

Whole-body fat mass was assessed by dual-energy X-ray absorptiometry (DXA) with a Prodigy ADV (Lunar Corp, WI, USA) adapted for assessment of children, delivering an irradiation dose of 0.1 mSv per assessment and having scanning precision CVs <3% [21]. Subcutaneous and visceral adipose tissue areas in the abdominal region were assessed by MRI using a multiple-slice MRI 1.5Tesla scan (Signa Excite HD, General Electric, Milwaukee, WI, USA). MRI was also used to assess liver fat, by comparing the intensity of the liver to that of subcutaneous fat and spleen, assuming that the latter organ is fat free [13]. The formula used was liver fat = 100 × (AIliver–AIspleen)/(AIadipose–AIspleen), wherein AI is average intensity. The DXA assessments and MRI scans were performed by the same operator and all images were analyzed by the same radiologist. Both specialists were blinded to treatment allocation.

### Data analysis

Results are expressed as mean ± SEM. Given the reconsideration into a pilot study no statistical analyses were performed. The percentage difference between metformin and placebo groups was assessed as the difference between the metformin percentage of change and placebo percentage of change. Percentage of change was assessed as the difference between the mean follow-up value and mean baseline divided by mean baseline and multiplied by 100 in metformin and placebo groups.

An intention-to-treat analysis was performed in our study. Every participant who had been randomized to either placebo or metformin was included in the analysis, ignoring noncompliance, protocol deviations, withdrawal, and anything that happened after randomization [25]. Data at 12 months were carried out at 24 months for those 6 participants who had dropped out from the study.

## Results

**Fig 1:**
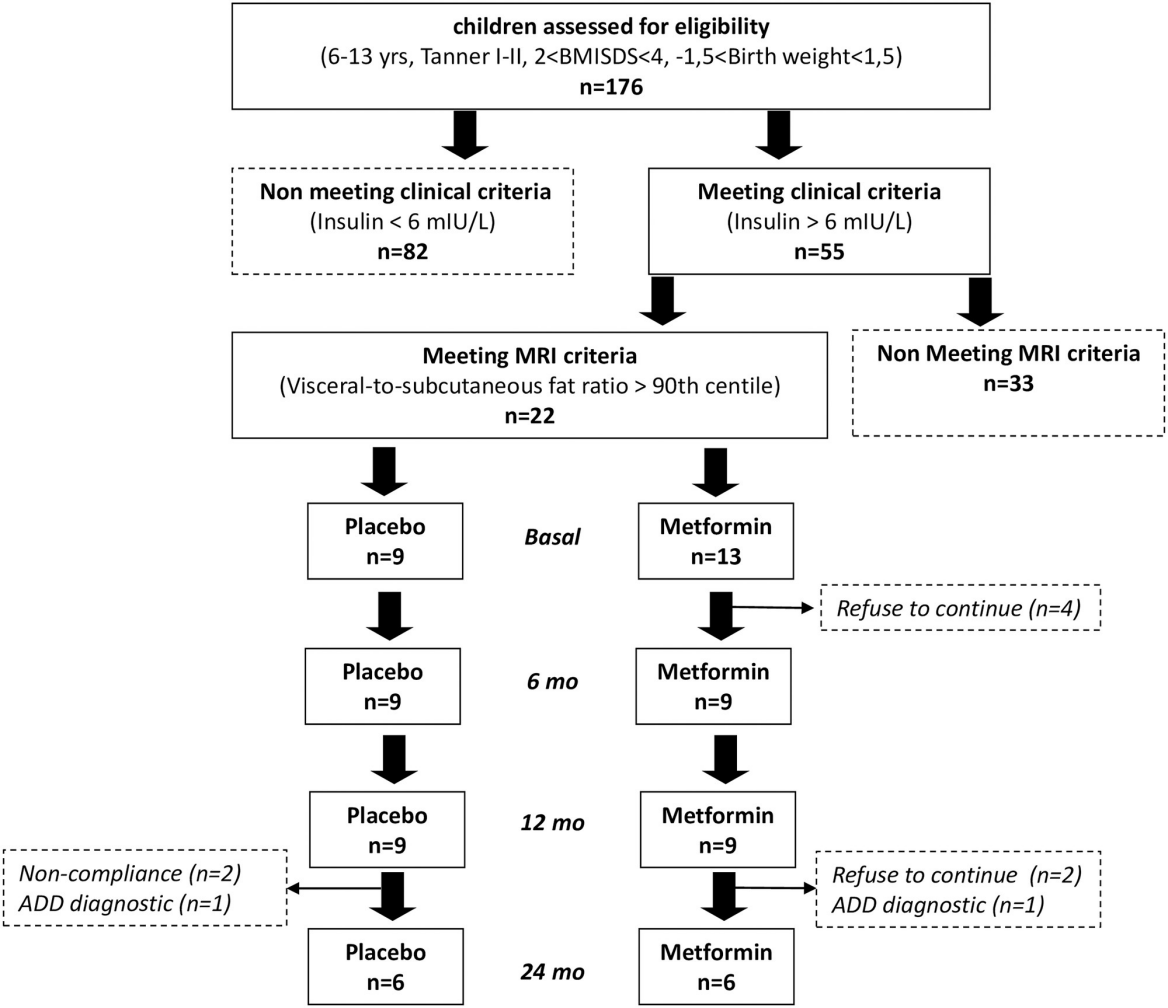
Recruitment of the study population. BMI, body mass index; AGA, adequate-for-gestational age; MRI, magnetic resonance imaging; ADD, attention deficit disorder.

Fig 1 shows the flowchart of the study population. From the 176 children assessed for eligibility 22 children met the clinical and MRI criteria to participate in the study and were randomized to receive placebo (n = 9) or metformin (n = 13). At start of the study, 4 participants in the metformin group refused to continue. From 12 to 24 months, 6 participants (3 participants in the metformin group and 3 participants in the placebo group) dropped out from our study because of noncompliance (n = 2), ADD diagnosis (n = 2), and refusal to continue in the study (n = 2).

Clinical, biochemical, and imaging data in the two randomized subgroups (placebo and metformin) at baseline and after 6, 12, and 24 months are shown in Table 1.

**Table 1:** Clinical, biochemical and imaging variables in the two randomized subgroups (placebo and metformin) at baseline and after 6, 12 and 24 months.

	0 mo		6 mo		12 mo		24 mo	
	Placebo (n = 9)	Metformin (n = 9)	Placebo (n = 9)	Metformin (n = 9)	Placebo (n = 9)	Metformin (n = 9)	Placebo (n = 9)	Metformin (n = 9)
Age (yr)	10.0 ± 0.5	8.8 ± 0.6	10.6 ± 0.5	9.3 ± 0.6	11.3 ± 0.5	9.9 ± 0.6	12.12 ± 0.6	10.9 ± 0.6
Sex (% female)	44	33	44	33	44	33	44	33
Tanner (stage)	1.2 ± 0.1	1.0 ± 0.0	1.4 ± 0.1	1.1 ± 0.1	1.8 ± 0.2	1.3 ± 0.2	2.7 ± 0.4	1.8 ± 0.4
Weight SDS	2.6 ± 0.2	2.9 ± 0.3	2.6 ± 0.2	2.4 ± 0.2	2.7 ± 0.3	2.2 ± 0.3	2.7 ± 0.2	2.5 ± 0.2
Height SDS	1.0 ± 0.2	1.1 ± 0.3	0.9 ± 0.3	1.0 ± 0.3	1.1 ± 0.3	0.9 ± 0.3	0.9 ± 0.3	0.9 ± 0.3
BMI SDS	2.6 ± 0.2	2.9 ± 0.2	2.7 ± 0.2	2.3 ± 0.2	2.7 ± 0.2	2.2 ± 0.2	2.7 ± 0.2	2.3 ± 0.2
Waist (cm)	86.5 ± 2.8	83.6 ± 2.8	87.8 ± 3.2	83.2 ± 3.6	89.2 ± 3.2	83.7 ± 3.5	91.3 ±2.9	86.5 ± 2.2
Glucose (mg/dL)	87 ± 2	90 ± 1	86 ± 1	90 ± 1	85 ± 2	87 ± 1	86 ± 2	88 ± 2
Insulin (mIU/L)	15.7 ± 3.6	10.1 ± 0.9	15.0 ± 2.0	11.6 ± 1.9	15.6 ± 1.7	13.4 ± 2.9	16.5 ± 2.1	14.2 ± 2.8
HOMA-IR	3.4 ± 0.8	2.2 ± 0.1	3.2 ± 0.5	2.6 ± 0.4	3.2 ± 0.3	2.9 ± 0.6	3.5 ± 0.5	3.2 ± 0.7
HDL-cholesterol (mg/dL)	46 ± 3	48 ± 3	48 ± 2	51 ± 3	44 ± 3	50 ± 3	44 ± 3	49 ± 4
Triglycerides (mg/dL)	94 ± 16	85 ±15	98 ± 14	94 ± 21	92 ± 9	85 ± 13	83 ± 7	77± 10
HMW-adiponectin (mg/L)	5.2 ± 0.9	5.3 ± 0.7	4.0 ± 0.7	5.3 ± 0.7	4.8 ± 0.9	5.8 ± 1.1	3.9 ± 0.8	5.2 ± 0.9
Leptin (ng/mL)	13.3 ± 3.7	17.5 ± 4.6	20.4 ± 4.1	14.1 ± 2.8	16.2 ± 3.3	11.8 ± 2.7	16.7 ± 3.8	11.4 ± 1.4
Leptin-to-HMWadiponectin ratio	4.3 ± 1.8	4.5 ± 1.8	7.5 ± 2.1	4.1 ± 1.6	6.8 ± 1.2	3.8 ± 1.0	6.2 ± 1.1	2.9 ± 0.7
hsCRP (mg/L)	3.1 ± 0.8	3.5 ± 1.1	3.4 ± 0.6	2.8 ± 0.9	3.2 ± 0.6	2.1 ± 0.7	3.1 ± 0.6	2.2 ± 0.7
cIMT (cm)	0.42 ± 0.02	0.44 ± 0.01	0.41 ± 0.01	0.42± 0.01	0.43 ± 0.02	0.38 ± 0.01	0.42± 0.01	0.38± 0.01
Fat Mass (kg)	25.5 ± 2.6	21.5 ± 2.9	27.6 ± 2.8	22.0 ± 3.0	29.8 ± 3.1	23.2 ± 2.9	31.7 ± 2.7	26.0 ± 2.5
Subcutaneous Fat (cm^2^)	200.8 ± 24.5	190.6 ± 23.6	226.8 ± 17.7	189.3 ± 25.6	243.7 ± 26.7	216.3 ± 31.6	241.8 ± 22.9	240.6 ± 27.2
Visceral Fat (cm^2^)	104.8 ±12.4	95.2 ±11.5	104.8 ± 10.5	95.1 ± 13.2	100.6 ±8.9	86.6 ± 8.8	102.0 ± 8.4	89.7 ± 8.6
Liver Fat (%)	14.8 ± 2.5	13.2 ± 2.2	13.1 ± 2.7	13.9 ± 1.9	16.4 ± 2.7	10.9 ± 1.0	16.8 ± 3.1	9.1 ± 1.0

Values are mean ± SEM. BMI: body mass index; SDS: standard deviation score; HOMA-IR: homeostasis model assessment insulin resistance; HMW-adiponectin: high molecular weight adiponectin; CRPus: ultrasensitive c-reactive protein; cIMT: carotid intima-media thickness; An Intention-to-treat analysis was preformed, including every participant who had been randomized. Data at 12 mo were carried out at 24 mo for those participants with missing data at the latter time point.

All participants were children born at term with normal birth weight for gestational age and a mean birth weight SDS of 0.5 ± 0.3. At baseline, nearly all were pre-puberal (Tanner stage 1.1 ± 0.1) with a mean age of 9.4 ± 1.8 years, with 39% girls. No differences in the studied variables between subgroups were observed at baseline.

**Fig 2:**
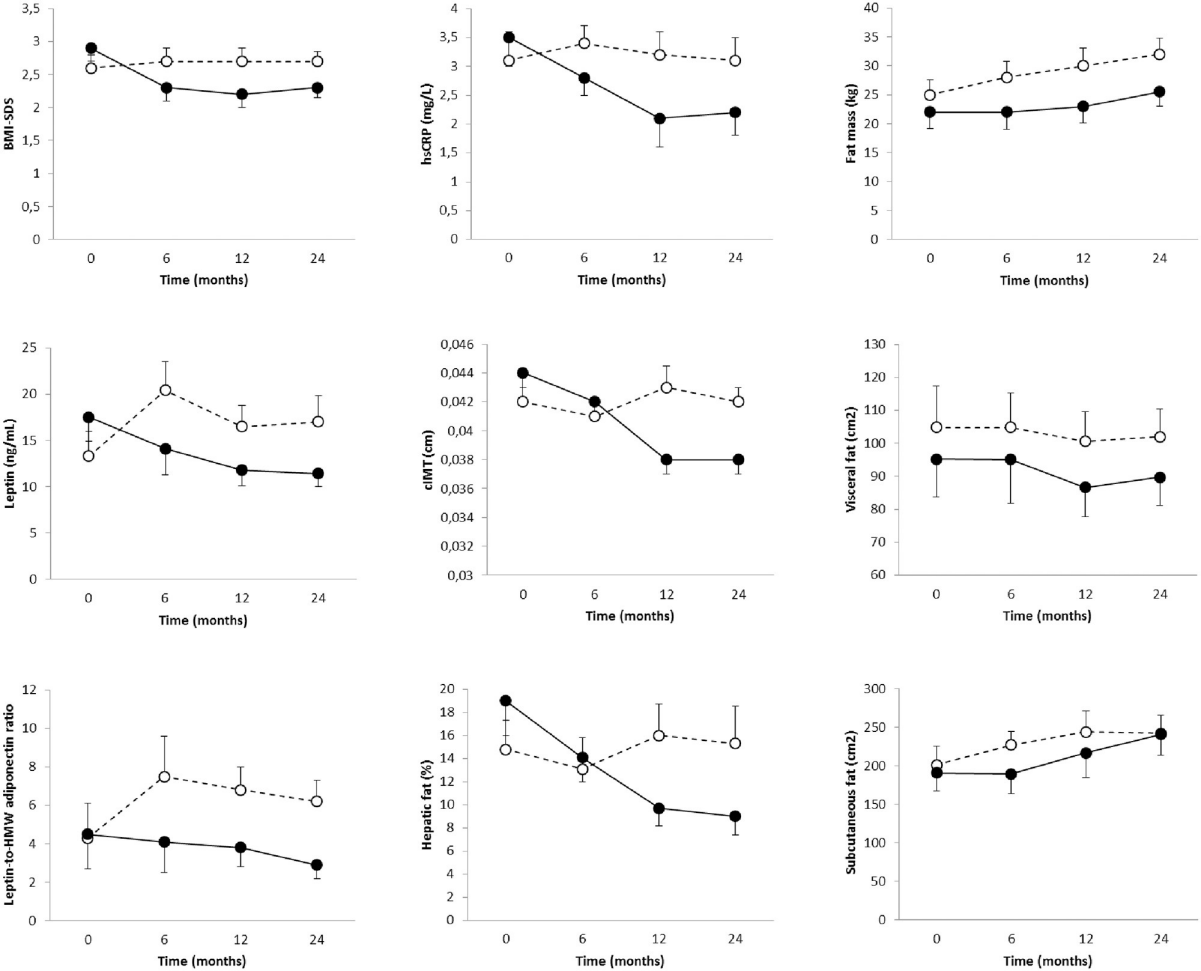
Differences in clinical, biochemical and imaging variables in the two randomized subgroups at baseline and after 6, 12 and 24 months.

After 6 months, a tendency of reduction in BMI-SDS (-0.6 ± 0.2 vs 0.1 ± 0.1), leptin (-3.4 ± 2.3 vs 7.1 ± 3.9), and leptin-to-HMW adiponectin ratio (-0.4 ± 0.5 vs 3.2 ± 1.1) was observed in children treated with metformin compared with those given placebo. The 12-month treatment seemed to cause a reduction in weight-SDS (-0.7 ± 0.2 vs 0.1 ± 0.1), BMI-SDS (-0.7 ± 0.2 vs 0.1 ± 0.1), leptin (-5.7 ± 2.7 vs 2.9 ± 4.3), leptin-to-HMW adiponectin ratio (-0.7 ± 0.7 vs 2.5 ± 1.2), CRPus (-1.4 ± 0.6 vs 0.1 ± 0.6), cIMT (-0.06 ± 0.01 vs 0.01 ± 0.01), fat mass (-0.8 ± 1.0 vs -0.2 ± 0.8), and liver fat (-2.3 ± 1.9 vs 1.6 ± 1.4) in children treated with metformin compared with those given placebo. The tendency of metformin in reducing BMI-SDS (-0.6 ± 0.2 vs 0.1 ± 0.1), leptin (-6.1 ± 3.6 vs 3.4 ± 4.3), leptin-to-HMW adiponectin ratio (-1.6 ± 1.5 vs 1.9 ± 1.3), CRPus (-1.3 ± 0.6 vs 0.0 ± 0.5) and liver fat (-4.1 ± 2.8 vs 2.0 ± 3.2) was maintained after completing 24 months of treatment. No changes in insulin sensitivity (HOMA-IR) or fasting insulin were detected throughout the study (Table 1 and Fig 2).

**Fig 3:**
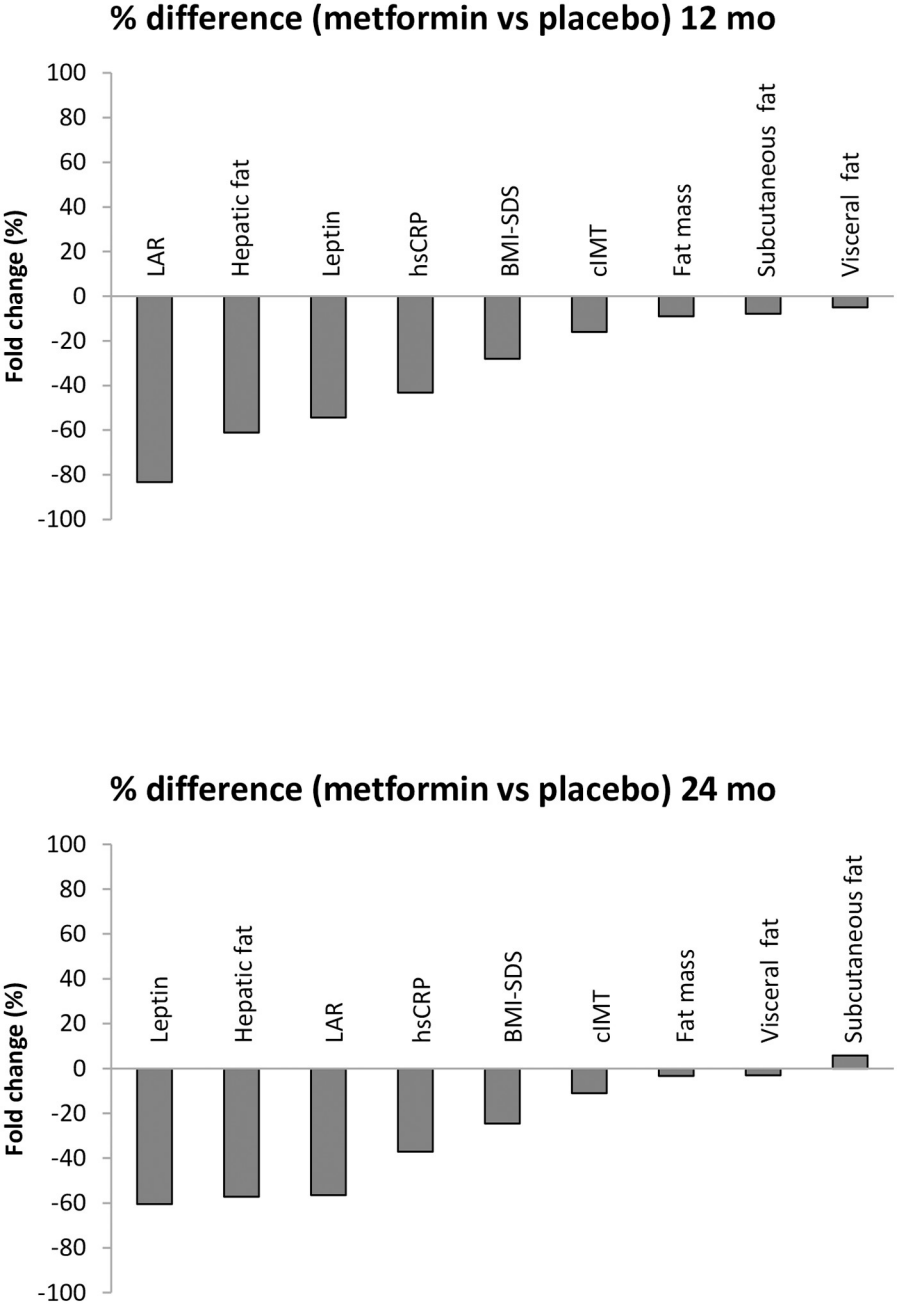
Percent difference in clinical, biochemical and imaging variables in metformin vs placebo group after 12 and 24 months. Negative values indicate a drop from placebo. LAR: leptin-to-HMWadiponectin ratio.

In terms of percentage of change, our results showed that metformin caused a reduction of 80% in leptin-to-HMW adiponectin ratio, 61% in liver fat, 54% in leptin levels, 43% in CRPus, 27% in BMI-SDS, 16% in cIMT, 9% in fat mass, 8% in subcutaneous fat, and 5% in visceral fat after 12 months (Fig 3). These percentages were maintained after completing the 24 months of treatment (Fig 3).

Metformin and placebo were well tolerated, and no side effects were reported. Pill counts at each study visit indicated that treatment compliance was good, except from 2 participants after 12 months of follow-up, who dropped out of the study because of lack of compliance.

## Discussion

In this pilot study, we enrolled pre-puberal and early pubertal children of both genders with obesity who had visceral fat excess and risk markers for metabolic syndrome in an attempt to explore new strategies to modify the associated metabolic risks over time by treating them with low-dose (850 mg) metformin over 24 months. Metformin intervention seemed to improve body composition, abdominal fat partitioning (decreased weight-SDS, BMI-SDS, fat mass, and liver fat accumulation), leptin levels, and leptin-to-HMW adiponectin ratio, and seemed to attenuate low-grade inflammation (decreased CRPus).

Several RCTs have already evaluated the beneficial effects of metformin in children with obesity and/or metabolic dysfunction; however, most of these studies were performed in pubertal children and adolescents and evaluated the effect of higher doses of metformin (1000–2000 mg/day) over shorter periods (6 months) [10, 15, 26–36] and only six studies had an intervention of more than 6 months [37–42]. Only one of those studies assessed the efficacy, in terms of weight loss and insulin resistance, of metformin at 12 and 24 months in children and adolescents with overweight and/or obesity; however, it was a retrospective study, with discrepancies in the metformin doses, and data from the metformin group were compared with a lifestyle intervention group [31].

One of the most beneficial effects described in all RCTs using metformin in children and adolescents is the significant decrease in BMI and/or weight. A BMI change of –1.3 kg/m^2^ and a change in weight of –3.9 kg were estimated in children treated with metformin compared with placebo over 6 months of treatment [6]. In this line, our results showed that metformin-treated children had a 25% decrease in BMI-SDS and a 20% decrease in weight-SDS compared with placebo-treated children over 12 months, which was maintained after 24 months. The metformin-induced decrease in BMI may thus reduce the risk factors for metabolic disease in these patients [43]. A reduction in leptin levels and in leptin-to-HMW adiponectin ratio was also observed after 6 to 24 months of treatment. Similar results have been reported by previous investigators in children with obesity [10, 27, 29, 39].

Metformin is likely to be also effective for cardiac and vascular disease prevention, as this drug improves endothelial function and provides protection from oxidative stress and inflammation [44, 45]. Several recently completed RCTs have reported effects of metformin on surrogate measures of atherosclerotic vascular disease, including cIMT, in people with type 1 (T1D) and type 2 (T2D) diabetes [46]. We report possible beneficial effects of metformin on cIMT reduction in children with obesity treated over 12 months.

We have also observed that metformin tended to attenuate low-grade inflammation by reducing hsCRP. Several studies in children evaluated this inflammatory biomarker after metformin interventions for 6 months, but none of them found significant changes [15, 27, 30, 32] probably because of the short-term treatment. However, previous studies have demonstrated beneficial effects of metformin on CRP in patients at high risk for diabetes after long-term intervention [47].

A few trials have assessed the effects of metformin treatment on body composition (by DXA) and abdominal fat (by MRI or abdominal computer tomography [CT]) with controversial results. Some of them found no statistically significant differences between groups in the percentage of body fat lost [32, 34, 35, 37] or abdominal fat content (by CT) [35]. One trial observed differences in total body fat in favor of the metformin-treated group, although changes in intra-abdominal fat (by MRI) were comparable between groups [38]. Conversely, two studies [27, 34] found a beneficial effect of metformin over placebo for subcutaneous abdominal fat but not for visceral abdominal fat (by MRI). We did not observe differences between groups in visceral or subcutaneous fat; however, although the percentage of subcutaneous fat increased 5% over the 24-month treatment, the percentage of visceral fat decreased by 3%. Moreover, our results showed that fat mass was significantly lower in metformin-treated children compared with placebo. These data suggest that the reduction in weight/BMI is likely to reflect a loss in fat mass, mainly in visceral fat.

In addition to abdominal fat, our results showed that metformin tended to reduce liver fat over 24 month of treatment. Among all RCTs of metformin in children, only one assessed liver fat and found no significant impact of metformin after 6 months, although there was a trend toward a lower percentage of liver fat over time [32]. In fact, we were not able to find differences between placebo and metformin-treated subgroups at 6 months, suggesting that the effect of metformin on liver fat could be observed only after a prolonged treatment.

Insulin sensitivity (as reflected by a decreased in fasting insulin and HOMA-IR) has been shown to be improved in children with obesity and hyperinsulinemia treated with metformin for 6 months [10, 15, 26, 27, 29, 31, 34, 35, 38]. Given metformin’s major effect in suppressing hepatic glucose production and in increasing peripheral glucose uptake in an AMPK-dependent manner [48], we expected to find a decrease in fasting insulin and HOMA-IR. However, our results failed to show an effect of metformin on insulin sensitivity. Similar results were obtained by others [28, 37]. The failure of metformin to significantly affect insulin sensitivity could be due to the low insulin-resistant status of our participants, as our children had relatively lower insulin levels than those published in other studies. Another reason could be the daily dose of metformin; we used 850 mg/day while the other studies used 1000–2000 mg/day. Moreover, we should note that significant insulin resistance typically emerges during puberty, so our children may have been too young to detect obvious changes with metformin.

Metformin therapy has been associated with several side effects [6]. Adverse events were reported to have occurred in 90% of the above-cited metformin trials in children with obesity using between 1000 and 2000 mg/day metformin. Gastrointestinal side effects were the most common, including diarrhea and abdominal pain. However, nausea, vomiting, upper respiratory tract infection, headache, migraine, fatigue, and musculoskeletal complaints have been also reported [10, 26, 30, 33, 34, 37, 38]. Srinivasan 2006 [34] reported that two participants in their study were unable to tolerate 1000 mg/day of metformin; however, they tolerated a lower dose and continued in the trial. Therefore, a change in dose or duration may solve these adverse effects [49]. As far as we know, this is the first study using 850 mg/day of metformin in children with obesity; this relatively low dose was well tolerated as none of the parents reported any adverse events during the course of the study in their children. Thus, our data suggest that a target total daily dose of 850 mg of metformin may have long-term cardiometabolic benefits and appears to be safe and well tolerated in young children with obesity and risk markers for metabolic syndrome.

This study has several limitations and strengths. The main limitation of our study was the small sample size, without enough power to detect significant differences, and the study was reconsidered into a pilot study. Consequently, a future fully powered trial to examine the use of low-dose metformin to improve metabolic abnormalities in obese children is needed. Another limitation was that after randomization, there were subtle differences between treatment groups in variables such us age, gender, and Tanner stage and we cannot rule out a possible effect of such variables in our results. Our study did not include a lifestyle intervention which could be also effective in improving the metabolic abnormalities in obese children. Further studies comparing metformin with lifestyle intervention are therefore warranted. The major strength of this study is the available data on metformin efficacy and safety in pediatric obesity. Moreover, we provide long-term data in a subset of pre-puberal and early pubertal children with obesity, that to our knowledge were unavailable so far in the literature, as most of the available trials were limited to 6 months.

Our results show that the use of low-dose metformin (850 mg/day) in young children with obesity and risk markers for metabolic syndrome is efficacious, well tolerated, and has potential long-term benefits in the improvement of body composition and inflammation markers. Our data encourage the development of future fully powered trials using 850 mg/day metformin in young children, highlighting its excellent tolerance and potential long-term benefits.

## Supporting information

S1 File CONSORT checklist. (PDF)

S2 File Study protocol. (PDF)
