# Supplementary material for: Effects of metformin administration on endocrine-metabolic parameters, visceral adiposity and cardiovascular risk factors in children with obesity and risk markers for metabolic syndrome: A pilot study
Source: PLoS One. 2019 Dec 10;14(12):e0226303. doi: 10.1371/journal.pone.0226303 (PMC6903728; doi:10.1371/journal.pone.0226303)
Supplement: S2 File — (PDF) [file pone.0226303.s002.pdf]

Clinical Trial Protocol

Title:
Effects on endocrine-metabolic parameters, visceral adiposity and cardiovascular risk factors of metformin administration in children with obesity and risk markers for metabolic syndrome

Protocol Code: **HJT-PEDOB-MET1** **Version 4 date: 21-05-12**

EudraCT: **2010-024414-61** Ministry of Science ICNC Project: **EC10-252**

Clinical Trial Sponsor:

Hospital of Girona
Dr. Josep Trueta Hospital
Av. Francia s/n
17007 Girona
Tel.: 972-940200
Fax: 972-485422

Principal Investigator:

Dr. Abel López Bermejo
Pediatrics.
Dr. Josep Trueta Hospital
Av. de Francia s/n
17600 Girona
Tel.: 972-940200. Ext. 2810
Fax: 972-485422

Trial supervisor:

Dr. Judit Bassols Casadevall
Pediatrics.
Dr. Josep Trueta Hospital
Av. de Francia s/n
17600 Girona
Tel.: 972-940200. Ext. 2810
Fax: 972-485422

APPROVAL AND SIGNATURES PAGE

Title:

EFFECTS ON ENDOCRINE-METABOLIC PARAMETERS, VISCERAL ADIPOSITY AND CARDIOVASCULAR RISK FACTORS OF METFORMIN ADMINISTRATION IN PREPUBERAL CHILDREN WITH OBESITY AND RISK MARKERS FOR METABOLIC SYNDROME

Protocol Code: **HJT-PEDOB-MET1** **Version 1 date : 13-Dec-10**

EudraCT: **2010-024414-61** Ministry of Science ICNC Project: **EC10-252**

We have read the protocol and agree to perform this clinical trial in accordance with all provisions defined in the current protocol according to Law 29/2006 that ensures the rational use of medicines and medical devices, the Royal Order 223/2004, which regulates the clinical trials with drugs, and the SCO Order 256/2007 on the application of rules for Good Clinical Practice. The Helsinki declaration (updated on 2008) and all applicable internacional legislations will be observed in this trial.

Principal Investigator and Coordinador of the clinical trial

Dr. Abel López Bermejo Signature _______________ Date __________

Approval. Medical Director, Hospital of Girona Dr. Josep Trueta

Dr. Rafael Masià Martorell Signature _______________ Date __________

Summary

1.1 Type of Study.

Phase II, randomized, double-blind, placebo-controlled clinical trial with parallel groups, one treated with metformin and the other with placebo as a control. All subjects included in the trial will be at risk of suffering metabolic syndrome, because they will have an abnormal fat distribution and endocrine-metabolic abnormalities (see inclusion criteria).
We aimed to study whether the treatment with metformin for 24 months is able to ameliorate the abovementioned alterations. The trial will end after a 6-month follow-up period without treatment.

1.2 Clinical Trial Sponsor:

Dr. Josep Trueta Hospital
Av. Francia s/n
17007 Girona
Tel.: 972-940200
Fax: 972-485422

1.3 Title:

EFFECTS ON ENDOCRINE-METABOLIC PARAMETERS, VISCERAL ADIPOSITY AND CARDIOVASCULAR RISK FACTORS OF METFORMIN ADMINISTRATION IN CHILDREN WITH OBESITY AND RISK MARKERS FOR METABOLIC SYNDROME.

1.4 Assay Code and EudraCT number:

Assay code: **HJT-PEDOB-MET1** **Version 4 date 21-05-12**
EudraCT: **2010-024414-61**
Ministry of Science ICNC Project: **EC10-252**

1.5 Principal Investigator

Dr. Abel López Bermejo
Pediatrics
Hospital of Girona Dr. Josep Trueta
Av. de Francia s/n
17600 Girona
Tel.: 972-940200. Ext. 2810
Fax: 972-485422
e-mail: alopezbermejo.girona.ics@gencat.cat

1.6 Trial Centers

Clinical Development

The clinical development of this assay will be multicentric and performed in the following hospitals:

1.- Hospital Dr. Josep Trueta de Girona

Investigador principal: Dr. Abel López Bermejo
Servicio de Pediatría
Hospital de Girona Dr. Josep Trueta
Av. Francia s/n
17007 Girona
Tel.: 972 940200
Fax: 972 940270
e-mail: alopezbermejo.girona.ics@gencat.cat

2.- Hospital de Santa Caterina (Parque Hospitalario Martí i Julià; Instituto de Assitencia Sanitaria), Girona

Investigador: Alex Suárez Berrio
Servicio de Pediatria
Edifici Santa Caterina, Parc Hospitalari Marti i Julià
Dr Castany s/n
17190 Salt
Tel.: 972 182500
Fax: 972 182575
e-mail: alexsuaolgabus@yahoo.com

3.- Hospital de Terrassa (Consorcio Sanitario de Terrassa), Barcelona

Investigadora: María Victoria Marcos
Unitat de Endocrinología Pediátrica
Consorci Sanitari de Terrassa
Hospital de Terrassa.
08027 Terrassa
Tfo.: 93 7003641
e-mail: maivemarcos@hotmail.com

Examinations and additional tests:

Examinations and additional tests will be carried out in the facilities of the hospital. Collaborations with external sites will be required if the hospital does not have the necessary facilities.

1.7 Clinical Research Ethics Comité.

We have requested the approval of the Ethics Committee on Clinical Research of Dr. Josep Trueta, as the reference Institutial Review Board.

All correspondence of this assay should be send to the reference IRB at the following address:

CEIC
Dr. Josep Trueta Hospital
Av. Francia s/n
17007 Girona
Tel.:972 940200
Fax: 972 940270
e-mail: ceic.girona.ics@gencat.cat

1.8 Name and qualification of the person responsible for trial supervision

Dra. Judit Bassols Casadevall
Pediatrics.
Dr. Josep Trueta Hospital
Av. de Francia s/n
17600 Girona
Tel.: 972-940200. Ext. 2810
Fax: 972-485422
e-mail: judit.bassols@gmail.com

1.9 Experimental and control drugs

1.9.1 Experimental drug:

The experimental drug is metformin (manufacturer: Kern Pharma SL), presenting as scored tablets containing 850 mg of metformin.

1.9.2 Control drug:

The control drug will be a placebo manufactured by the same company: Kern Pharma SL.

1.10 Clinical Trial phase

Phase II/III. It will test the use of metformin as a treatment of children with prepuberal obesity and markers of metabolic syndrome, in order to prevent the development of metabolic syndrome and its related vascular and endocrine-metabolic abnormalities.

1.11 Objetives

Primary:

1) To ascertain if metformin treatment for 24 months normalizes the endocrine-metabolic abnormalities, improves body composition –specifically, decreases the excess of visceral fat and intrahepatic lipidic content-, and reduces the carotid intima-media thickness in prepubertal obesity.

2) To ascertain if prepubertal girls with obesity, unfavorable body composition and risk markers for metabolic syndrome treated with metformin for 24 months will show a delayed onset of puberty compared to placebo-treated girls;

Secondary:

1) To identify prepubertal children with obesity, unfavorable body composition, and risk markers for metabolic syndrome;

2) To ascertain the efficacy and safety of long-term metformin use in prepubertal children with obesity.

1.12. Study Design

Phase II/III, randomized, double-blind, placebo-controlled clinical trial with parallel groups for the study of a new indication of metformin in the treatment of endocrine-metabolic disorders secondary to childhood obesity and risk markers for metabolic syndrome, including abnormal fat distribution, specifically, an increase in intraabdominal visceral fat.

1.13. Disease of interest

Obesity (defined as body mass index above the percentile 97) affects 9% of children according to a Spanish Health Survey in 2006. Despite the large increase in the prevalence of obesity in children, the therapeutic control of the disease is very limited. Onset of childhood obesty is commonly seen at the time of the adipose rebound at or after 6 years of age. Early development of childhood obesity is commonly associated with markers for metabolic syndrome. Furthermore, prepubertal obese girls may develop an early and rapidly progressing puberty with impaired final height. Early treatment with metformin in prepubertal children with obesity and risk factors for metabolic syndrome may prevent and/or ameliorate these endocrine-metabolic abnormalities and improve the long term prognosis of the disease. The current proposal, based on previous work from our research group, is the first placebo-controlled randomized trial in prepubertal obese children.

1.14 Study population and total number of patients

Subjects will be Caucasian children (n=44) aged 6.0 to 12.9 years, of both sexes, with obesity, unfavourable body composition and risk markers for metabolic syndrome.

1.15 Patient Selection

The patients will be recruited at the referral areas of all participating hospitals: 1) at the pediatric primary care clinics; 2) by phone or letter, according to computerized records of the health area; a first appointment will be given for a preselection visit.

On this first contact, subjects will be informed about the characteristics of the study and the initial inclusion criteria will be reviewed, including:

- ⇒ the most recent current auxological data
- ⇒ auxological data at birth (weight, height and gestational age)

Information provided on this first contact has been approved by the Ethics Committee (See below section 10 “ethical aspects”).

At the preselection visit, which will be performed within 30 days after the initial contact, the abovementioned auxological criteria will be reviewed again. Informed written consent will be obtained at this visit. After signing the informed consent, patients will be appointed for the next selection visit (see diagram) about 15 days before the enrollment visit or trial start. Laboratory tests and additional examinations will be appointed according to the diagram below.

At the selection, the results of the laboratory tests and additional examinations will be reviewed to verify the compliance of all the inclusion criteria and absence of all of the exclusion criteria. If necessary, additional examinations will be requested to confirm that all the data are complete on the day of the enrollment visit.

Phases of trial

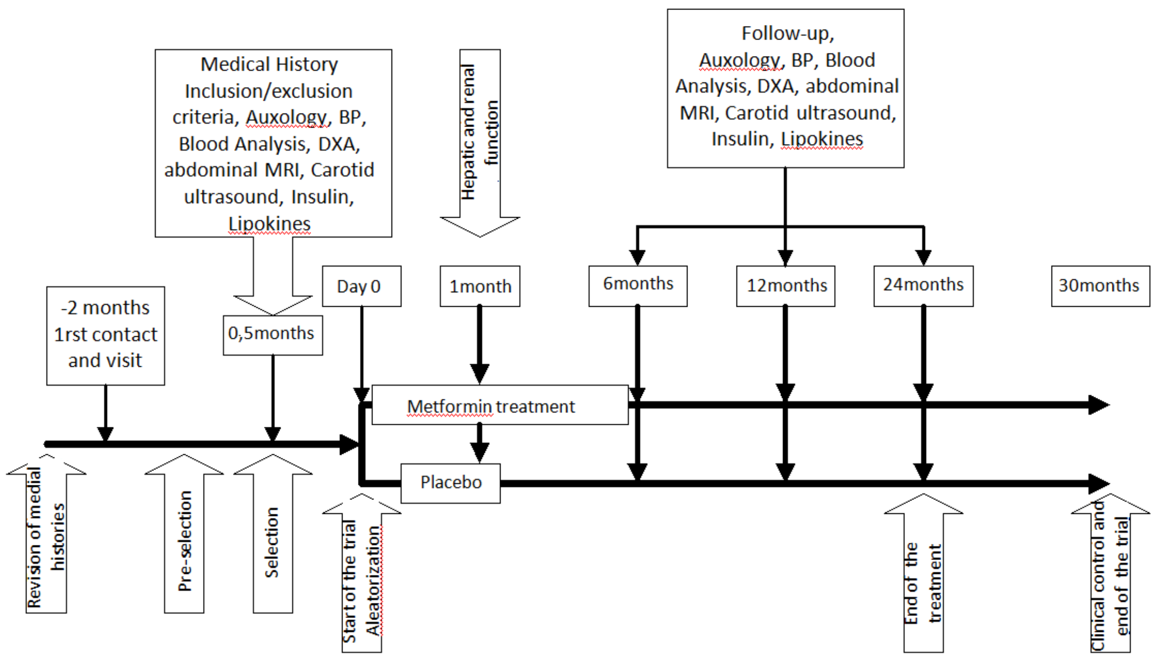

A group (n=22; treatment group) will receive metformin at a dose of 850mg/day and the other group (n=22, control group) will receive placebo during 24 months. Both groups will be balanced by blocks according to sex and age.

1.15 Inclusion and exclusión criteria

Inclusion criteria (all must be fulfilled):
1) Caucasian children aged 6.0 to 12.9 years;
2) Body mass index [BMI, between 2 SD (97th centile) and 3 SD, for age and sex];
3) Visceral fat area [magnetic resonance (RM)] > 90th centile, based on a reference of healthy children without obesity;
4) Visceral-to-subcutaneous fat ratio (RM) > 90th centile, for the same reference of healthy children;
5) Baseline insulin > 90th centile, for healthy children;
6) Birth weight above -1.5 SD and below +1.5 SD for gestational age, to avoid the influence of birth weight deviations on metabolic and cardiovascular risk markers;
7) Absence of puberty or initial stages of puberty (Tanner<4; breasts up to Tanner 3 for girls and testicles up to 10 ml in boys).
The BMI of the patient must be stable (along the same percentile) for the last 3 months prior to potential inclusion in the trial.

Exclusion criteria (any of them):
1) Drug or alcohol abuse during gestation;
2) Genetic syndromes;
3) Hypothalamic obesity;

At the time of inclusion:
4) Abnormalities in thyroid, hepatic or renal function or in serum electrolytes;
5) Known skin allergies;
6) Glucose intolerance or type 2 diabetes;
7) Chronic illnesses other than obesity;
8) Treatment with corticosteroids, sexual hormones, and drugs that may alter glucose tolerance or insulin sensitivity;
9) Acute infections or use of anti-inflammatory drugs or antibiotics 2 weeks prior to potential inclusion in the study;
10) Medical treatment or other therapies aimed at reducing body weight (3 months prior to potential inclusion in the study).

1.16 Treatment Duration

After a screening period of 8 weeks, a randomization will be performed according to the procedure available at the hospital pharmacy, in groups of 2 or 3 patients, two for each gender, age group and treatment arm to complete a total of 44 subjects. The randomization table will be not visible to the researchers until the end of the evaluation.

Potential candidates to participate in the trial will be scheduled for a preselection visit at the hospital within 30 days of the initial contact. The family will be asked to provide retrospective auxological data of the patient. On this visit, the family will have to sign the informed consent, and the the auxological inclusion criteria of the subject will be reviewed (weight, height and current body mass index, and weight and length at birth).
Laboratory tests (blood tests, including glucose and insulin), and radiological examinations (magnetic resonance imaging to assess visceral adiposity) will be performed if the patient meets all the auxological criteria to determine whether he or she meets the remaining inclusion criteria. These tests will be performed no later than 2 weeks from the date of the initial visit.

The decision on whether or not include the patient in the study will be done no later than 2 weeks after completing the laboratory tests and radiological examinations. If the patient happens to be a candidate for the trial, a new measurement of height and weight will be performed to calculate body mass index. On this visit, the randomization (metformin or placebo) will be done at the Hospital Pharmacy. The family will collect the medication on the same day. Subjects will therefore commence the study no later than 2 months after the first contact with the family.

The recruitment of subjects will last 12 months. Definite inclusion in the study will occur on the first day of treatment, which will span 24 months for each patient. A preliminary data analysis will be done after half of the patients have completed 12 months of the treatment period, which will allow us to detect the existence of qualitative and/or quantitative differences between groups. Follow-up visits and additional examinations will be made at 0, 6, 12 and 24 months on treatment. The trial will be end after a 6-month follow-up period without treatment.

1.17 Variables

The present clinical trial includes the analysis of pathophysiological aspects of obesity and also of the response to treatment and of prognostic variables, so it will include a wide range of parameters:

Primary endpoints of the study:
Insulin sensitivity (HOMA), insulin, visceral and liver fat, visceral-to-subcutaneous fat ratio, and carotid IMT. We will consider as positive and discriminative responses an increase of more than 30% in insulin sensitivity (estimated by the HOMA method), a decrease of more than 15% in fasting insulin, a decrease of at least 10% in visceral or liver fat or in the visceral-to-subcutaneous fat ratio, and a decrease of at least 15% in carotid IMT.

Secondary variables: onset of puberty (girls), adipocytokines.

Contextual variables:
- Clinical: auxology (weight, height, waist and hip circumferences), systolic and diastolic blood pressure, bone age [Greulich & Pyle6], data on nutritional and physical activity (validated questionnaires for Spanish children) at inclusion in the study. Perinatal data and information regarding familiar history for metabolic and cardiovascular diseases will also be collected.

- Endocrine-metabolic parameters: glucose, insulin, IGF-I, lipid profile, adipokines (high molecular weight adiponectin, leptin, omentin and ultrasensitive C reactive protein). All these parameters are analyzed on a routine basis at our centre.
- Body composition: DXA values for bone mineral density and bone mineral content measured in absolute and in relative values, total lean and fat mass, trunk fat mass, abdominal fat mass. Abdominal fat measured by MRI: subcutaneous and visceral fat mass and intrahepatic lipid content.
- Vascular: intima-media thickness in both carotid arteries (high-resolution ultrasound).

1.18 Schedule and expected date of completion.

The expected duration of this trial is 3 years. Once the project is approved by the Ethics Committee and the Competent Authority the assay will start, approximately in July 2011, and will be completed in December 2014.

- Scheduled start date: July 2011.
- Estimated completion date: December 2014.
- Duration of the inclusion period: 12 months.
- Total duration of the assay: 24 months from the start of treatment
- Duration of the post-treatment observation period: 6 months.

1.19. Assay development and evaluation of the response

The summary of the major milestones are outlined in the following table:

Parameter	basal	6 m	12 m	24 m	6 m post
Weight, height, BMI	X	X	X	X	X
Blood pressure	X	X	X	X	X
Bone age	X		X	X	
Blood count, lipid profile, liver and renal function	X	X	X	X	
Thyroid function	X		X	X	
Glucose, insulin, IGF-I	X	X	X	X	
Adipokines	X	X	X	X	
Body Composition (DXA)	X	X	X	X	
Visceral fat and IHLC (abdominal MRI)	X	X	X	X	
Carotid IMT (Doppler ultrasound)	X	X	X	X	

1.20 Data Analysis

Statistical analyses will be performed using SPSS version 12.0 (SPSS Inc, Chicago, IL). Differences in quantitative variables between treatment groups -before, during and after treatment- will be analysed by general lineal models for repeated measurements adjusting for possible confounding variables. Intention-to-treat analysis and analysis of patients with full data will be done at 6, 12, and 24 months of treatment. Significance will be set al $p<0.05$.

1.21 Ethical Aspects

The protocol will be carried out in accordance with the Declaration of Helsinki updated in October 2008 and the Spanish legislation regarding clinical trials in children. A written informed informed consent will be obtained from parents or children's guardians and the public prosecutor will be informed with each new inclusion of a participant. The study protocol will be approved by the Institutional Review Board for Clinical Research of the participating centers and by the Spanish Agency of Medicines and Health Products (Ministry of Health).

1.22 Practical Aspects

Both metformin and placebo will be supplied by the Pharmacy of the participating institutions, where a record of all supplied and returned medication will be kept. On each visit, the patients will receive the required medication until the next visit to the hospital. Participants must provide the empty boxes of medication dispensed at the previous visit.

Protocol Index **Page**

1. **Summary** 3
2. **Index** 11
3. **General Information** 14
3.1. Assay Identification 14
3.2. Type of trial 14
3.3. Description of the studied products 14
3.4. Trial sponsor 15
3.5. Trial supervisor 15
3.6. Trial investigators 15
3.7. Trial centers 17
3.8. Expected duration of the trial 17
4. **Justification of the study and objetives** 17
4.1. State of the art and justification 17
4.2. Objectives of the clinical trial 21
5. **Type of clinical trial and design** 22
5.1. Type of clinical trial 22
5.2. Design and randomization. Sampling process 22
6. **Patients selection** 25
6.1 Number of patients 25
6.2 Inclusion criteria 25
6.3 Exclusion criteria 26
6.4 Enrollment period 26
6.5 Criteria for trial withdrawal and analysis of withdrawals and dropouts 27
7 Treatment overwiev 27
7.1 Description of the doses, intervals and route 27
7.2 Regime changes 30
7.3 Criteria for dose modification 30
7.4 Drug toxicity 30
7.5 Measures to be taken in case of overdose 30
7.6 Concomitant incompatible treatments 31
8 Trial development and evaluation of response 31
8.1 Pre-selection and start of study 31
8.2 Description of experimental period 32
8.3 Primary endpoint 33
8.4 Secondary variables 33
8.5 Description of the variables 34
8.6 Flowchart 39
9. Adverse events 39
9.1 Definitions 39
9.2 Information of adverse events occurring in a patient during the clinical trial 40
9.3 Accountability critera 40
9.4 Procedures for immediate notification of serious or unexpected adverse events 40
9.5 Reporting of adverse events 40

10 Ethical aspects 40
10.1 Clinical trial insurance 40
10.2 Subject identification 41
10.3 Patient information 41
10.4 Consent form 41
11 Practical considerations…..41
11.1 Specific rules for patients and familiars 41
11.2 Administrative issues 41
11.2.1 Compliance and revisions of the protocol 42
11.2.2 Trial supervision 42
11.2.3 Documents and reports 42
11.2.4 Relations with the Clinical Research Ethics Comite 42
11.2.5 Policy of publications 42
11.3 Product under research……………..……………………………………..……….. 42
11.3.1 Product identification 43
11.3.2 Management and delivery 43
11.3.3 Packaging and labeling 43
11.3.4 Supply application 43
11.3.5 Deposit of the research product 43
11.3.6 Return of the research product 43
11.3.7 Destruction of the research product 43
12 Statistical analysis 44
12.1 General plan 44
12.2 Descriptive statistics and data evaluation 44
13 References 44

APENDIX Pag.

Apendix 1 – Glossary of acronyms…………………………………………………….….... 47
Apendix 2 – Declaration of Helsinki ………………………….……………………… 48
Apendix 3 – Reporting of adverse events…... ………………...……………………… 53
Apendix 4 – Neonatal standard tables ……………..…………………………………… 54
Apendix 5 – Growth charts …………….……………………………………………… 55

3. General Information

3.1 Assay Identification

Title:

EFFECTS ON ENDOCRINE-METABOLIC PARAMETERS, VISCERAL ADIPOSITY AND CARDIOVASCULAR RISK FACTORS OF METFORMIN ADMINISTRATION IN PREPUBERAL CHILDREN WITH OBESITY AND RISK MARKERS FOR METABOLIC SYNDROME

Protocol Code: **HJT-PEDOB-MET1** **Version 4 date: 21-05-12**

EudraCT: **2010-024414-61** Ministry of Science ICNC Project: **EC10-252**

3.2 Type of trial

Phase II/III, randomized, double-blind, placebo-controlled clinical trial with parallel groups, one treated with metformin and the other with placebo as a control. All subjects included in the trial will be at risk of suffering metabolic syndrome, because they will have an abnormal fat distribution and endocrine-metabolic abnormalities (see inclusion criteria).

We aimed to study whether the treatment with metformin for 24 months is able to ameliorate the abovementioned alterations. The trial will end after a 6-month follow-up period without treatment. Indedd, we wish to confirm a new therapeutic indication for metformin, according to the properties described later in this protocol, based on the experience of the research group and on available bibliographic references.

The experimental treatment will be compared with placebo because the current evidence, although supportive of the use of metformin in obese children, is still insufficient and clinical trials providing definitive conclusions have not been yet carried out.

3.3 Description of the study products

The product to be tested is metformin tablets (manufacturer: KERN pharma SL). It is made as white scored tablets containing 850 mg of metformin. The brand name is that for the generic product in addition to that for the manufacturer and has the national authorization number 652220 and registry number 67066. This preparation has been chosen because the tablets are easy to break into two pieces for a better use in pediatrics.

The therapeutic indications of metformin correspond to those of the oral hypoglycemic biguanides group, i.e., type 2 diabetes mellitus, particularly in overweight patients, when the specific diet and exercise are not enough to normalize blood glucose. In adults, it can be used alone or in combination with other oral agents or insulin. In children, it is accepted above the age

of 10 years and in adolescents, alone or in combination with insulin. The code that corresponds to the anatomotherapeutic clasification is the oral antidiabetic ATC A01BA02.

The placebo will be prepared by Kern Pharma SL with the corresponding legal and regulatory approvals. It will be dispensed in identically labeled boxes with the same appearance as the metformin tablets by the pharmacy of the participating centers, without being able to make any distinction. The list of the contents and numbers of boxes will be under custody at the pharmacy and will be kept secret until the trial finishes, except eventual emergency.

Other details related to the products used in this study are provided in paragraph 7.

Treatment Overview

3.4 Data related to clinical trial Sponsor

The Clinical Trial Sponsor of this trial is:

Dr. Josep Trueta Hospital
Av. Francia s/n
17007 Girona
Tel.: 972-940200
Fax: 972-485422

This is a second-order university hospital with an integrated service of pediatrics.

3.5 Trial supervisor

The supervisor Head will be:

Dr. Judit Bassols Casadevall
Pediatrics
Dr. Josep Trueta Hospital
Av. de Francia s/n
17007 Girona
Tel.: 972-940200. Ext. 2810
Fax: 972-485422

3.6 Trial investigators

Principal Investigador

The principal investigator and Head of the trial will be:

Dr. Abel López Bermejo
Pediatrics
Dr. Josep Trueta Hospital
Av. de Francia s/n
17007 Girona
Tel.: 972-940200. Ext. 2810
Fax: 972-485422
e-mail: alopezbermejo.girona.ics@gencat.cat

Research Team

The research team consists of the research personnel at the reference hospital: Dr. Josep Trueta Hospital (see 3.7 center where the clinical trial will be performed).

The research team consists of Dr. Abel López Bermejo and a physician to be hired by the project that is responsible for conducting the clinical trial with the inclusion and examination of patients, and the collection and analysis of the data.

The trial includes the collaboration of two additional hospitals: Hospital at Terrassa (Consorci Sanitari de Terrassa, Barcelona) and Santa Caterina Hospital (Parc Hospitalari Martí i Julià; Institut d'Assitència Sanitària, Girona). The following pediatric endocrinologists at these centers: Dr. Alejandro Suárez and Dr. Maria Victoria Marcos, will participate with the inclusion and follow-up of subjects.

Other centers involved in the project are: primary care centers at Taiala and Montilivi, where Dr. Rosa Cortes, Dr. Emilio Fortea and Dr. Cristóbal Buñuel will participate with the recruitment of patients who will be referred to Dr. Josep Trueta Hospital for potential inclusion in the study and follow up.

The pediatric department at Dr. Josep Trueta Hospital, with Dr. Cristina Casas, Dr. Montserrat Gispert-Saucha and Dr. Luis Mayol, as well as the Pediatric Department of Hospital of Figueres, with Dr. Ines Osiniri, will also collaborate with ultrasound studies and data analysis.

The abovemention ultrasound experts have the necessary tools and experience to perform the carotid vascular studies.

Patients' examination and laboratory test will take place at the hospital of reference, according to the standards of quality specific from the hospital. Dr. Judit Bassols, Anna Prats, Dr. Mercè Montesinos and Dr. Pilar Soriano, are specialists in particular determinations of the trial, specifically in the assessment of adipokines (total adiponectin and high molecular weight adiponectin, leptin, omentin and C-reactive protein), which will be held at the Hospital of Girona and at the Research Laboratory of the Institute of Biomedical Research of Girona.

The researchers demonstrate previous experience in conducting similar studies and clinical trials in pediatric patients. The principal investigator is a senior investigator funded by the I3 program from Ministry of Science and Innovation.

Radiological studies and other examinations will be made at the following centers:

• Clinica Girona (Dr. Patricia Reyner and Dr. Joan Carles Vilanova): DXA and RM of abdomen.

These centers have ample experience in these techniques and have the necessary equipment to carry out the studies on body composition and quantification of visceral fat and intrahepatic lipic content.

3.7 Centers where the clinical trial is performed

The coordination of the trial will be carried out at the Department of Pediatrics, Dr. Josep Trueta Hospital, with the participation of the following center:

1.- Dr. Josep Trueta Hospital (reference hospital)

Principal Investigator: Dr. Abel López Bermejo
Pediatric Endocrinology
Hospital Dr. Josep Trueta
Avda. Francia s/n
17007 Girona
Tel.: 972 940200
Fax: 972 940270
e-mail: alopezbermejo.girona.ics@gencat.cat

Collaborating Centres:

1.- Clínica Girona

Investigadores colaboradores: Dra. Patricia Reyner y Dr. Joan Carles Vilanova
Servicios de DXA y de RM
Clinica Girona
C. Joan Maragall 26
17002 Girona
Tel.: 972 010300
Fax: 972 206666

3.8 Expected duration of the trial

The expected duration of this trial is 2 years for each patient from the start of treatment. Upon granting all mandatory authorizations (local IRB and the Spanish Agency of Medicine and Medical Devices, as competent authority), the study will be ready to start, which is expected within the first half of year 2011, in order to finish by the end of year 2014, including an observation period following treatment of six months.

4. Justification of the study and Objectives

4.1 State of the art and Justification

Obesity is an important problem of public healthy owing to the exponential increase of its prevalence in recent years (1); indeed, the prevalence of pediatric obesity has tripled in one decade (2). Obesity in an independent risk factor for metabolic (dyslipidemia, type 2 diabetes, metabolic syndrome) and cardiovascular diseases (hypertension, atherosclerosis).

Metformin is a biguanide widely used to treat type 2 diabetic patients (see below). It has been successfully used to reduce the increase in weight, the hyperinsulinism and dyslipidemia in these

patients and, in prediabetic subjects, to prevent the progression from glucose intolerance to type 2 diabetes (3-4). These results have prompted the use of metformin in adolescents in the early phases of development of diabetes and metabolic syndrome, mainly in those subjects with obesity and hyperinsulinism, in order to reduce the risk for metabolic and cardiovascular diseases (5). Recently, it has been reported that metformin treatment over 6 months in adolescents with obesity and hyperinsulinism was associated with a 1.42 kg/m2 reduction in body mass index (BMI) and a 2.01 unit decrease in the insulin resistance index (HOMA-IR) (6).

Our recent studies in prepubertal girls with a history of low birthweight (LBW), increased visceral adiposity and risk markers for metabolic syndrome show that treatment with metformin before and throughout puberty ameliorates the increase in total, visceral and hepatic adiposity, and prevent the deterioration of endocrine-metabolic parameters (7). In these girls, metformin treatment was followed by a delayed progression of puberty, a more normal menarcheal age and a higher adult height (8, 9).

Puberty, together with prenatal and early postnatal life, are highly dynamic periods that are characterized by a plasticity of the epigenome to adapt to the increased metabolic and growing demands of these phases of life (10). Our previous studies in prepubertal girls without obesity but with an increase in visceral adipocity and cardiometabolic risk markers show that prepubertal metformin treatment not only prevents the worsening of the metabolic profile in these girls, but has persistent effects beyond this period of life, once the pharmacological treatment has been discontinued (11). Although a postpubertal onset of metformin treatment also improves the metabolic profile in these girls, metformin's normalizing effects wear off as soon as the drug is discontinued (12). It has therefore been proposed that puberty offers a window of opportunity for reprogramming, through epigenetic changes, the metabolic abnormalities of these subjects in order to delay a potential development of metabolic syndrome (11).

- **Scientific and public health relevance of the project:**

We herein describe a clinical trial that analyzes, for the first time, the effects of early metformin treatment on the metabolic abnormalities that are associated with pediatric obesity. The analysis of the short-term effects of metformin will be followed by an extension of the assay throughout all the pubertal period (approximately 4 years) to analyze and compare, for the first time to our knowledge, the effects of early versus late onset of metformin therapy.

Considering the results from our previous studies and the data supporting a plasticity of the epigenome during the pubertal period, we hypothesize that early treatment with metformin (before puberty or initial stages of puberty) is essential to ensure its efficacy and long-lasting effects (9). The long-term beneficial effects of metformin would be no longer present if therapy is started after puberty.

The prevalence of obesity is reaching alarming proportions in our country (2).The metabolic and cardiovascular comorbidities that result from obesity impose high economic burdens in nearly all developed societies (13). This project will investigate if treatment with metformin before onset of puberty or initial stages of puberty, as compared with treatment early postpuberty, is more effective in terms of normalization of cardiometabolic risk markers in obese children. The project opens a new perspective in the prevention of obesity-associated metabolic syndrome.

- **Justification of the project:**

Despite the marked increase in the prevalence of pediatric obesity, the therapeutic control of this disease has been elusive. Metformin has proven to be efficacious in the reduction of metabolic and cardiovascular disease risk in adult subjects and has an excellent safety profile. Metformin is also included in the priority list for studies into off-patent pediatric medicinal products by the European Union (7th Framework Program; Annex 1). However, little is known about the efficacy of this drug in obese children. Metformin has been mainly used in obese pubertal or postpubertal adolescents (6). To the best of our knowledge, only one study has been reported in a mixed population of pre and postpubertal children (see below).

Our studies in prepubertal girls without obesity, but with increased visceral adiposity and cardiometabolic risk markers, show that pubertal metformin results in an effective and long-lasting normalization of the impaired metabolic profile in these patients. Prepubertal onset of metformin treatment was essential to ensure its efficacy and long-lasting effects (9). It is therefore timely to similarly investigate the effects of metformin treatment in obese prepubertal children and in initial stages of puberty.

The study population will be school-aged (prepubertal and initial stages of puberty) obese children (BMI higher than 97th centile) with risk markers for metabolic syndrome. Given a prevalence of obesity of around 9%, according to a recent survey in Spain (year 2006), it is expected that the results of this study will have a high impact in terms of population health.

- **Justification of the lack of commercial interest in the proposal:**

Metformin was marketed in Europe three decades ago, but it was not approved in USA until 1994. It is likely the most commonly used prescription drug worldwide for type 2 diabetes. No patent exists on the production or use of metformin to prevent or treat type 2 diabetes or metabolic syndrome and the active substance is marketed as a low-cost generic drug.

- **Studies related with the clinical trial:**

Metformin is an insulin sensitizer (14), widely tested in adults, adolescents and pregnant women. Metformin has been widely used to treat type 2 diabetic patients, and is currently approved for this indication in patients aged 10 or older. It has an excellent safety profile, short, medium and long term, not only in adolescents with diabetes, but also in nondiabetic patients who have insulin resistance associated entities and hyperinsulinism.
As described previously, metformin has proven effective in improving cardiovascular and metabolic profile in obese adolescents (6).

Below are detailed studies related to the current proposal:

<u>Active clinical trials:</u>
NCT00005669: Effects of Metformin on Energy Intake, Energy Expenditure, and Body Weight in Overweight Children With Insulin Resistance. Country: USA. Development phase: active. Start year: 2000. Study population: children and adolescents aged 6 to 12 years old with obesity and hyperinsulinism (n=277). TREATMENT: metformin or placebo for 6 months. Main outcomes: changes in BMI and body composition measured by DXA.

NCT00120146: A Multi-Center, Randomized, Placebo Controlled, Double- Blind Trial of Metformin in Obese Adolescents. Country: USA. Development phase: active. Start year: 2002. Study population: adolescents aged 13 to 17 years of age with obesity (n=22). TREATMENT: metformin or placebo. Main outcomes: changes in BMI.

NCT00139477: Understanding the Effects of Therapeutic Intervention on Cardiovascular Risk Markers, Insulin Resistance, and Intra-Hepatic Fat Contents in Obese Children at High Risk for the Metabolic Syndrome. Country: USA. Development phase: active. Start year: 2005. Study population: children and adolescents aged 8 to 18 years with obesity (n=160). TREATMENT: metformin or placebo. Nutritional intervention and exercise. Main outcomes: biomarkers of metabolic syndrome.

Active clinical trials in which the IP collaborate:
ISRCTN58810841: Endocrine-metabolic and body composition effects of metformin administration in prepubertal children with a low birthweight for gestational age, postnatal catch-up growth, and risk markers for metabolic syndrome: a double-blind randomised placebo-controlled trial. Country: Spain. Development phase: active. Start year: 2009. Study population: prepubertal children 6 to 9 years with a history of low birth weight and risk markers for metabolic syndrome (n=52). TREATMENT: metformin or placebo for 24 months. Main outcomes: changes in insulin sensitivity and visceral fat.

ISRCTN19548431: Effects of metformin on cardiovascular risk factors in prepubertal children born small for gestational age without postnatal catch-up growth, currently treated with growth hormone: a prospective randomised clinical trial. Country: España. Developmental phase: active. Start year: 2010. Study population: prepubertal children 6 to 9 years old with a history of low birth weight who are treated with growth hormone (n=64). TREATMENT: metformin or placebo for 12 months. Main outcomes: changes in insulin sensitivity and visceral fat.

Recently finished:
ISRCTN43267711: Insulin resistance and obesity in adolescents - metabolic characterisation and effects of therapeutic intervention with metformin. Country: Australia. Developmental phase: Finished. Start year: 2005. Study population: children and adolescents 9 to 18 years old with obesity and hyperinsulinism (n=unknown). TREATMENT: metformin or placebo.

ISRCTN19517475: Metformin in Obese Children with Abnormal Glucose and Insulin Status MOCA. Country: Reino Unido. Developmental phase: finished. Start year: 2005. Study population: children and adolescents 9 to 18 years old with obesity and hyperinsulinism (n=80). TREATMENT: metformin or placebo. Main outcomes: changes in BMI.

Results of clinical trials published metformin and childhood obesity:

Atabek ME, Pirgon O. Use of metformin in obese adolescents with hyperinsulinemia: a 6-month, randomized, doubleblind, placebo-controlled clinical trial. J Pediatr Endocrinol 2008;21:339–348.

Srinivasan S, Ambler GR, Baur LA, Garnett SP, Tepsa M, Yap F, Ward GM, Cowell CT. Randomized, controlled trial of metformin for obesity and insulin resistance in children and adolescents: improvement in body composition and fasting insulin. J Clin Endocrinol Metab 2006;91:2074–2080

Freemark M, Bursey D. The effects of metformin on body mass index and glucose tolerance in obese adolescents with fasting hyperinsulinemia and a family history of type 2 diabetes. Pediatrics 2001;107: E55

Love-Osborne K, Sheeder J, Zeitler P. Addition of metformin to a lifestyle modification program in adolescents with insulin resistance. J Pediatr 2008;152: 817–822

Yanovski JA, Sorg RA, Krakoff J, Kozlosky M, Sebring NG, Salaita CG, Keil M, Mc- Duffie JR, Calis KA. A randomized, placebo- controlled trial of the effects of metformin on body weight and body composition in children with insulin resistance. Abstract presented at the 90th Annual Meeting of The Endocrine Society, 16 June 2008, Moscone Center, San Francisco, California

<u>See meta-analysis based on the results of these 5 clinical trials showing efficacy of metformin for improve the metabolic and cardiovascular profile in obese adolescents:</u>

Park MH, Kinra S, Ward KJ, White B & Viner RM. Metformin for obesity in children and adolescents: a systematic review. Diabetes Care 2009 32 1743–1745.

<u>Last published trials:</u>

Wiegand S, l'Allemand D, Hubel H, Krude H, Burmann M, Martus P, et al. Metformin and placebo therapy both improve weight management and fasting insulin in obese insulin-resistant adolescents: a prospective, placebo-controlled, randomized study. Eur J Endocrinol 2010;163:585-92.

Wilson DM, Abrams SH, Aye T, Lee PD, Lenders C, Lustig RH, et al. Metformin extended release treatment of adolescent obesity: a 48-week randomized, double-blind, placebo-controlled trial with 48-week follow-up. Arch Pediatr Adolesc Med 2010;164:116-23.

4.2 Objetives of the clinical trial

This clinical trial not only studies the effects of metformin on endocrine-metabolic alterations in obese children, but because of the methodology used, is a cohort study to analyze the clinical and endocrine-metabolic changes over time of these patients. Thus we can define the main objectives directly related to the treatment effect and secondary objectives, related to the follow up of obese children, which may allow to better define the disease under study and the safety of the treatment.

Primary objectives:

1) To ascertain if metformin treatment for 24 months normalizes the endocrine-metabolic abnormalities, improves body composition –specifically, decreases the excess of visceral fat and intrahepatic lipidic content-, and reduces the carotid intima-media thickness in obesity.

2) To ascertain if prepubertal girls with obesity, unfavorable body composition and risk markers for metabolic syndrome treated with metformin for 24 months will show a delayed onset of puberty compared to placebo-treated girls.

Secondary objetives:

1) To identify children with obesity, unfavorable body composition, and risk markers for metabolic syndrome.

2) To ascertain the efficacy and safety of long-term metformin use in prepubertal children with obesity.

5. Type of clinical trial design.

5.1. Type of clinical trial

This clinical trial protocol results from the adaptation required by the Standards of Good Clinical Practice (Order SCO 256/2007 of 5 February) of the project financed by the Ministry of Health No. EC10-252, under the call for independent clinical research (BOE-A-2010-14036 September 11). This protocol has been approved by the local IRB and the Spanish Agency of Medicines and Health Products.

This is a Phase II/III clinical trial, where the treatment with metformin or placebo will be used in 44 children (prepuberty and initial stages of puberty) with obesity and risk factors for metabolic syndrome, with abnormalities of body composition (increased visceral adiposity). We aimed to study whether metformin can prevent or delay a further metabolic deterioration and to characterize all the changes.

5.2. Design and randomization, sampling process

The subjects will be 44 obese patients aged 6.0 to 12.9 years divided in equal groups of 22 males and 22 females. To ensure that the selection of the sample is random and is balanced by age, sex and treatment group, metformin or placebo, the following steps will be carried out:

a) Development of a list of candidates to be preselected:

Prior to patient selection, a list of patients will be generated according to the following criteria (see inclusion criteria):

1.- Obesity: body mass index [BMI, ≥2SD(97th centile) and ≤3SD for age]
2.- Normal weight and height at birth (above -1.5 SD and below +1.5 SD for gestational age)
3.- Results from additional examinations suggesting a increased risk of evolving into a metabolic syndrome.

A list with candidates will be prepared using the databases of the health area of the participating centers, for a total of 44 patients divided into groups of 22 boys and 22 girls. Each group of 22 will be subdivided into 4 age groups, according to sex and birth year, from 2001 to 2004, arranging them in order in tabular form.

In this way, we will select 2 or 3patients of each sex and age group, which once informed they will be scheduled for a preselection visit, with cross-check of inclusion and exclusion criteria, and clinical examination and radiological exams.

During the preselection visit, candidates will be informed of the study (see section 10 of ethics). If the patient cannot be contacted or he or she does not meet all the inclusion and exclusion criteria, the candidate will be replaced by another patient chosen from the same sex and age block (sampling with replacement).

In this way, we will have balanced blocks of 10 patients according to sex and age, who will be randomly assigned to each of the two treatment arms. Each step of selection will be registered together with the reasons for including or not the patients. Once it has been cross-checked that all the prelected subjects meet all the inclusion and exclusion criteria, they will be assigned a sequential trial number that will remain constant for each patient and will be used as the identification number for data report and other documents of the trial.

b) List of candidates for selection and randomization of treatment groups. Sampling with replacement

By the selection visit, all the inclusion and exclusion criteria will have been cross-checked in all eligible subjects. During this process some patients may be lost if they do not meet the abovementioned criteria, or if they are not willing to accept to participate for other reasons. Should this happen, a replacement should be made, ie, if the preselected patient can not be included, the verification process will be repeated in the remaining patients of the same sex and age group who have not been selected yet. As the patients are being included, an assay number will be assigned to each subject test. The assay treatement will be randomly assigned according to the following double-entry table, where patients remain in 8 blocks of 10 subjects balanced for age, sex and treatment.

The 44 patients, male and female, for the purposes of sampling, can be distributed according to a table like the following, where MT means male patients treated, FT female patient treated, MC control male and FC control female. According to this table there are available 44 randomized positions, 22 for boys and 22 for girls, 11 subjects balanced for treatment, age (birth year) and sex. A1 in the table means the first year, A2 second, the second year, and so on:

	Boys	Girls
Treated	Block MTA1 of 3 p.<br>Block MTA2 of 2/3 p.	Block FTA1 of 3 p.<br>Block FTA2 of 2/3 p.<br>.
Controls	Block MCA1 of 3 p.<br>Block MCA2 of 2/3 p.	Block FCA1 of 3 p.<br>Block FCA2 of 2/3 p.

In the following chart, a description of the sampling and inclusion processes are given:

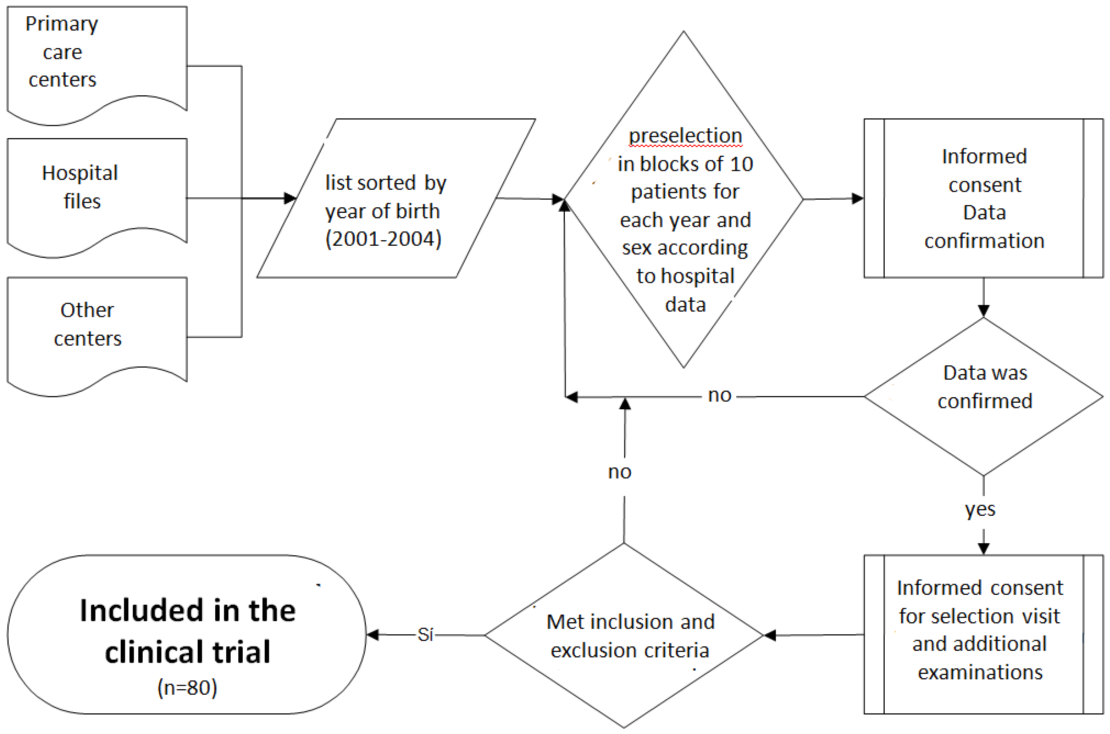

Diagram of sampling

Once a random list has been generated after the preselection process and all the inclusion and exclusion criteria have been verified, the subjects will be ready for a selection visit. Upon signing the informed consent, subjects will be randomly assigned to each of the treatment arms. Note that this process is strictly confidential and can only be known by the pharmacist responsible for giving out the medication. It can only be disclosed for a patient in case of an emergency or if it is necessary for his or her treatment.

The pharmacist responsible for giving out the medication is also responsible for compliance with the randomization tables as shown above. Neither the patients, nor the research will be allowed to know to which treatment arms the patients are assigned.

From the beginning, both treatment groups will followed in parallel without any external distinction.

6. Patient selection

The trial will include 44 patients, aged between 6.0 and 12.9 years, prepubertal or initial stages of puberty (Tanner<4), of both sexes, with obesity and risk markers for metabolic syndrome. Patients will be selected in the area of influence of the participating hospitals: 1) at the primary care centers, 2) by letter. They all will receive detailed information about the trial. A written informe consent will be obtained from the parents and, when applicable, assent from the children. Patients born between 1st January 2000 and 31st December 2005 who are considered as per protocol possible candidates will enter in a pre-selection phase.

6.1. Number of patients

In our previous studies in adolescents with a history of low-birth weight, precocious puberty, hyperinsulinism, subclinic hyperandrogenism and dyslipidemia (who show increased visceral adiposity in prepuberty), metformin treatment resulted in improved insulin sensitivity (HOMA), decreased insulin levels and a reduction in abdominal fat mass. Relative to placebo, insulin sensitivity increased by 38%, insulin levels decreased by 18%, and abdominal fat mass decreased by 13%, on metformin over 12 months. Consequently, if we wish to detect (with a potency of 80% and a significance level of $p<0.05$) differences similar to those herein stated in insulin sensibility or in the gain in visceral fat between the metformin- and the placebo-treated groups, a total of 56 patients will be needed per group. An estimated final number (n=44 per group) will be included in order to account for potential patient dropouts on follow up (which has been estimated to be up to 30%).

6.2 Inclusion Criteria

Each patient must fulfil every one of the following criteria to be selected to participate in the study:

1) Body mass index [BMI, between 2 SD (97th centile) and 3 SD, for age and sex] (16)
2) Visceral fat area [magnetic resonance (RM)] > 90th centile, based on a reference of healthy children without obesity (16)
3) Visceral-to-subcutaneous fat ratio (RM) > 90th centile, for the same reference of healthy children (16)
4) Baseline insulin > 90th centile, for healthy children (16, 17)
5) Birth weight above -1.5 SD and below +1.5 SD for gestational age (15), to avoid the influence of birth weight deviations on metabolic and cardiovascular risk markers.
6) Absence of puberty or initial stages of puberty (Tanner<4; breasts up to Tanner 3 for girls and testicles up to 10 ml for boys). (18,19)
The BMI of the patient must be stable (along the same percentile) for the last 3 months prior to inclusion in the trial.

6.3 Exclusion Criteria

A patient may not be included in the study if he or she meets any of the following exclusion criteria:

1) Drug or alcohol abuse during gestation;
2) Genetic syndromes;
3) Hypothalamic obesity
At the time of inclusion:
4) Abnormalities in thyroid, hepatic or renal function or in serum electrolytes;
5) Known skin allergies;
6) Glucose intolerance or type 2 diabetes;
7) Chronic illnesses other than obesity
8) Treatment with corticosteroids, sexual hormones, and drugs that may alter glucose tolerance or insulin sensitivity;
9) Acute infections or use of anti-inflammatory drugs or antibiotics 2 weeks prior to potential inclusion in the study;
10) Medical treatment or other therapies aimed at reducing body weight (3 months prior to potential inclusion in the study).

6.4 Enrollment period

Potential candidates to be included in the study will be referred to the centers where the trial will be carried out and, after obtaining the informed consent, the doctors will verify the details of their medical history and additional examinations will be performed to confirm that they meet all of the inclusion and exclusion criteria. The phases of this process have been previously described (section 5.2.b):

a) The potential candidates to be included in the trial will be recruited among those ascertained from the medical records and databases in the participating centers and will be randomly pre-selected after receiving information by mail. They must show their interest in the study. These patients will be scheduled for a first contact visit at the participating centres within 30 days of their assentment. The family will be asked to provide retrospective auxological data of the patient and its reliability will be cross-checked, especially with regard to neonatal data (pediatric health book or pediatrician report with information on birth history data.)

b) On this visit, the family will sign the informed consent upon agreement with the study, and the inclusion auxological criteria (weight, height and current body mass index and weight and length at birth) will be cross-checked.

c) If the patients meet the auxological criteria, he or she will be scheduled for the laboratory tests and additional examinations needed to determine whether he/she meets the additional criteria of the trial (glucose, insulin, and magnetic resonance imaging to assess visceral adiposity). These tests will be performed within 2 weeks after the initial visit.

d) The results of the laboratoy and radiological examinations will be assessed and the decision of whether or not the patient can be included in the study will be made within a

maximum of 2 weeks after performing of the tests. If the patient happens to be a candidate for trial, a new measurement of height and weight and calculation of body mass index will be performed. In this visit, the patient will be randomized to either metformin or placebo and teh family will collect the test medication on the same day.

e) Therefore, the maximum time between the first contact with the family and the beginning of the study will be 2 months. (See sampling chart 5.2b)

For details see section **8. Trial development and evaluation of response**.

It is expected that the inclusion of 44 patients will be performed in one year. After the selection period the trial will last two years from the start of treatment, according to the schedule below:

- Scheduled start date: July 2011.
- Estimated completion date: December 2014.
- Duration of the inclusion period: 12 months.
- Total duration of the assay: 24 months from the start of treatment
- Duration of the post-treatment observation period: 6 months.

6.5 Criteria for trial withdrawal and analysis of withdrawals and dropouts

The experience of the research team (20, 21) suggests that the treatment will be well tolerated and there will be few dropouts. If the event the patient wishes to leave the trial or any adverse event occurs that makes it necessary to discontinue the participation in the study, the patient will be requested to come to the end-of-study visit. If necessary, the adverse-event document will be filled-in and dispatched.

In the analysis of results, both the patients who have completed the study and the dropouts will be considered in an intention-to-treat analysis. An analysis as per protocol will be also performed and the differences between the two methods will be studied (see section **12. Statistical analyses**). Each case of lost on follow-up or dropout will be assessed individually, in order to justify all actions taken.

A list of all patients who were unwilling or unable to be included in the trial will be kept. These patients will be asked, once the test finished, to come to a visit with the investigators in order to discuss the possible incidences on health that may have occurred during the trial dates, which will serve as a reference for the trial results.

7. Treatment overview

7.1 Description of the doses, intervals, administration routes and forms

The experimental treated group will receive 1 tablet a day of Metformin Kern Pharma SL ® as a single oral administration, corresponding to 850 mg of drug.

The description of the product characteristics can be obtained from the technical and official prospectus that can be downloaded from the web www.agemed.es in the paragraph of Human Health Professionals, Data Sheet Drugs, registration code 64927.

Some of the features are herein summarized, which cannot replace a thorough reading of the related technical papers.

Composition and dosage form

The pharmaceutical form consists on white, elongate, biconvex tablets, bilaterally scored, containing 850 mg of metformin hydrochloride, which corresponds to 662.9 mg metformin base. The composition of the excipients and the coating film of the tablet are contained at the technic data sheet.

Mechanism of action

Metformin is a hypoglycemic biguanide that does not stimulate insulin production, so it rarely causes hypoglycemia. Its biological actions are known to invove three mechanisms:

⇒ Reduction of hepatic glucose production through a reduction in gluconeogenesis and glycogenolysis
⇒ Increasing insulin sensitivity and increasing glucose uptake in peripheral tissues, especially in muscle
⇒ Delaying intestinal glucose absorption

It is considered that metformin increases the transport capacity of all types of membrane glucose transporters (GLUT). It has also favorable effects on lipid metabolism.

Pharmacokinetic properties

Absorption after an oral dose of metformin (500 to 800 mg) reached Tmax at 2.5 hours with a bioavailability of 50-60% and an unabsorbed fraction detectable in feces ranging from 20 to 30%.

With usual doses, plasma metformin concentrations are attained within 24 to 48 hours and usually are lower than 1 μg /mL. In controlled clinical trials, peak plasma levels (Cmax) do not exceed the 4 μg/mL, even with maximum doses. Food delays absorption and decreases its bioavailability by 25%.

Metformin binds little to the plasma proteins and enters the erythrocytes. The average volume of distribution is very variable, ranging between 63 and 276 L.

It is excreted unchanged in the urine; no metabolites were detected in human. The renal clearance is 400 mL/min, which means that it is eliminated by filtration and tubular secretion. In normal subjects, an oral dose has an elimination half-life of 6.5 hours.

Safety

With a normal kidney function and if there are no interactions with other products or processes, metformin is well tolerated and hypoglycemia is not observed even with doses up to 85g.

Overdose or concomitant risks can trigger a lactic acidosis that requires hospitalization. Hemodialysis is the procedure of choice to remove lactate and metformin in case of poisoning.

Adverse reactions

⇒ Common gastrointestinal symptoms (> 10%), nausea, vomiting, diarrhea etc. that usually subside within a few days, especially if treatement is started with low doses.
⇒ Frequent metallic taste (3%)
⇒ Erythema secondary to hypersensitivity (<0.01%)
⇒ Decrease in the absorption of vitamin B12 in chronic treatments, without clinical relevance (<0.01%)
⇒ Rare lactic acidosis (0.03 cases/1000 patients/year), more commonly seen in case of dehydration, kidney insuficiency, drug interactions or other causes.

Special precautions must be taken in pregnancy and lactation, which is not the case in this trial.

Treatment with metformin at doses up to six times higher than those intended to be used in this study has no negative effects on liver and kidney function, does not alter serum electrolytes, vitamin B12 and folic acid or cause lactic acidosis.

The finding of blood glucose under 3.58 nmol/L (65mg/dL), and a progressive increase in the levels of creatinine and/or urea, transaminases, accompanied or not by suggestive symptoms, will cause the discontinuation of treatment.

Likewise, the existence of signs and/or suggestive symptoms of hypoglycemia, the onset of a persistent rash, the presence of abdominal pain, nausea and/or vomiting, headaches, sinusitis, severe bacterial infections, even when not accompanied by alterations in the laboratory tests will also cause the discontinuation of treatment.

In cases where it is necessary to stop treatment, renal and liver function and blood glucose levels, will be monitorned until the disappearance of symptoms or normalization of the altered parameters.

Directions

Metformin is used for the treatment of type 2 diabetes mellitus in children above 10 years when diet and exercise do no suffice to offset the high blood sugar.
Treatment must be stopped before surgery and should not be resumed within the initial 48 hours. Interactions with iodinated contrasts and alcohol should be avoided. Caution with concomitant use of diuretics, IECAs, glucocorticoids and beta2 agonists must be exerted (See data sheet or treaties of pharmacology)

Pediatric clinical practice

In the context of clinical practice, metformin is considered as an insulin sensitizer, widely tested in adults, children, adolescents and pregnant women (20-23). It inhibits hepatic glucose production and increases peripheral tissue sensitivity to insulin. It also inhibits the synthesis of adrenal and gonadal androgens, and promotes bone formation by inducing the differentiation and

mineralization of osteoblasts (22, 24). Metformin has been used for years in the treatment of type 2 diabetes and is currently approved for this indication in patients of 10 years or more. It has an excellent safety profile, at short, medium and long term, not only in children with diabetes, but also in nondiabetic patients who have clinical syndromes associated with hyperinsulinism and insulin resistance (15, 20-23).

Dose justification

Treatment with metformin at doses up to six times higher than those intended to be used in this study has no negative effects on liver and kidney function, does not alter serum electrolytes, vitamin B12 and folic acid or cause lactic acidosis.
However, the finding of blood glucose levels below 3.58 nmol/L (65 mg / dL), and a progressive increase in serum creatinine and/or urea, transaminases, with or without suggestive symptoms will indicate the discontinuation of treatment.

Likewise, the existence of signs and/or suggestive symptoms of hypoglycemia, the onset of a persistent rash, the presence of abdominal pain, nausea and/or vomiting, headaches, sinusitis, severe bacterial infections, even when not accompanied by alterations in the laboratory tests will also indicate the discontinuation of treatment.
In cases where it is necessary to stop treatment, renal and liver function and blood glucose levels, will be monitorned until the disappearance of symptoms or normalization of the altered parameters.

7.2 Regime changes
7.3 Criteria for dose modification

These two sections are not pertinent to this trial. The dosage used is low and it is not expected to have short-term effects, but rather long-term effects. Therefore there are no data to suggest the possibility of modifing the doses or criteria for doing so. If during the trial there are novel findings to suggest these possibilities, they will be taken into account.

7.4 Drug toxicity
7.5 Measures to be taken in case of poisoning

Metformin is usually well tolerated in apparently-healthy subjects. Caution should be exerted in cases of ketoacidosis or diabetic patients who with altered sensorium. Caution is also warranted in allergic patients experiencing reactive dermatitis, in whom the treatment would be the same as for allergic reactions.
Most worrisome is the possibility of lactic acidosis, which requires adequate rehydration and alkalinization measures in the intensive care unit. This condition has only been reported in elderly diabetics with abnormal renal function. The use of hemodialysis has been noted above. It should be added that such reactions are unlikely at the doses used in this study.

7.6 Concomitant incompatible treatments

Painkillers such us paracetamol are allowed.

Incompatible medications: anticoagulants, anti-inflammatory drugs, including corticosteroids, oral hypoglycemic agents, antiandrogens, estrogens, progestagens, antibiotics in general and especially vancomycin, trimethoprim/sulfamethoxazole and trimethoprim, cimetidine, ranitidine, iodinated contrasts, some of them because they interfere with the action of metformin and some because they modify the variables of interest for the trial.

Before prescribing any medication, the advice of the investigators should be sought, the event should be written down in the case report form and the patient should be considered to be removed from the trial.

8. Trial development and evaluation of response

The added value of this trial is double, I) on one side, it is a cohort of children (prepubertal or initial stages of puberty) of both sexes, from 6.0 to 12.9 years, suffering from a disease that may hinder their future development and is recognizable by a specific clinical pattern at baseline (inclusion criteria) and II) the initial cohort will be randomly divided into two test groups, one of whom will receive treatment and the other placebo, which will allow to compare the treatment effect not only on the initial alterations in the patients, but on the features of the parallel changes of the two groups.

8.1 Pre-selection and start of study

Patients pre-selected for the study must exhibit the following characteristics:

1) Body mass index [BMI, between 2 SD (97th centile) and 3 SD, for age and sex] (16)
2) Birth weight above -1.5 SD and below +1.5 SD for gestational age (15), to avoid the influence of birth weight deviations on metabolic and cardiovascular risk markers.
3) Absence of puberty or initial stages of puberty (Tanner <4; breasts up to Tanner 3 for girls and testicles up to 10 ml for boys). (18,19)

And, before their final inclusion in the study, that they must also show the following risk markers for metabolic syndrome:

1) Visceral fat area [magnetic resonance (RM)] > 90th centile, based on a reference of healthy children without obesity (16)
2) Visceral-to-subcutaneous fat ratio (RM) > 90th centile, for the same reference of healthy children (16)
3) Baseline insulin > 90th centile, for healthy children (16, 17)

These alterations must be considered in the context of the adipose tissue as an endocrine organ, which not only acts as an energy reservoir to buffer changes in caloric intake over time, but it is an endocrine organ producing a number of endocrine and paracrine molecules and an effector of hormonal mediators and neurotransmitters. The diagram below depicts the role of adipose tissue as an endocrine organ and several of the adipose-derived substances.

Some of these substances are exclusively synthesized in subcutaneous and/or visceral adipose tissue; others are secreted by macrophages (Figure). The adipokines may act either as mediators or markers that define an inflammatory or anti-inflammatory, procoagulant or atherogenic state or may influence the hypothalamus and gonadal systems (26-28).

The substances above outline three patterns to be considered in the development of the trial:

⇒ **Alteration** of normal development
⇒ **Preliminary alterations** suggestive of risk
⇒ **Context** of humoral changes, hormones, adipokines, mediators of inflammation, etc., that will be prospectively analyzed

This is depicted in the following figure and gives support to the schedule of the trial that is discussed below.

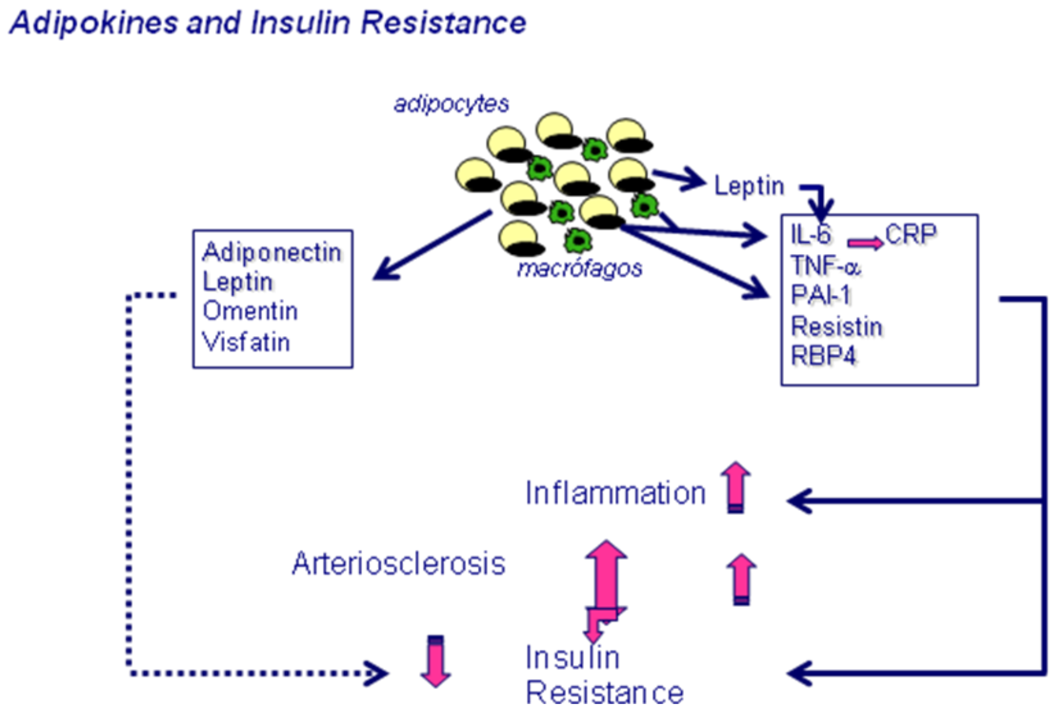

8.2 Description of experimental period

The changes expected in this clinical trial involve slow occurring modifications, so the initial determinations, defining the baseline state, will be compared with the values recorded at 6, 12 and 24 months. An auxological evaluation will be carried out also at 30 months, 6 months after the end of treatment, as a safety measure. The number of parameters to assess is complex and can be summarized in the following points:

⇒ Clinical: auxology (weight, height, waist and hip circumferences), systolic and diastolic blood pressure, bone age [Greulich & Pyle (29)], data on nutritional and physical activity

(validated questionnaires for Spanish children) at inclusion in the study. Perinatal data and information regarding familiar history for metabolic and cardiovascular diseases will also be collected.

⇒ Endocrine-metabolic parameters: glucose, insulin, IGF-I, lipid profile, adipokines (high molecular weight adiponectin, leptin, omentin and ultrasensitive C reactive protein). All these determinations are carried out on a routine basis at our centre.

⇒ Body composition: DXA values for bone mineral density and bone mineral content measured in absolute and in relative values, total lean and fat mass, trunk fat mass, abdominal fat mass. Abdominal fat measured by RM: subcutaneous and visceral fat mass and intrahepatic lipid content (30)

⇒ Vascular: intima-media thickness in both carotid arteries (high-resolution ultrasound).

Determinations

Parameters	basal	6 m	12 m	24 m	6 m post
Weight, height, BMI	X	X	X	X	X
Blood pressure	X	X	X	X	X
Bone age	X		X	X	
Blood count, lipid profile, liver and renal function	X	X	X	X	
Thyroid function	X		X	X	
Glucose, insulin, IGF-I	X	X	X	X	
Adipokines	X	X	X	X	
Body Composition (DXA)	X	X	X	X	
Visceral fat and IHLC (RM abdomen)	X	X	X	X	
IMT carotid (high resolution ultrasound)	X	X	X	X	

8.3 Primary endpoint

Insulin sensitivity (HOMA), insulin, visceral and liver fat, visceral-to-subcutaneous fat ratio, and carotid IMT. We will consider as positive and discriminative responses an increase of more than 30% in insulin sensitivity (estimated by the HOMA7 method), a decrease of more than 15% in fasting insulin, a decrease of at least 10% in visceral or liver fat or in the visceral-to-subcutaneous fat ratio, and a decrease of at least 15% in carotid IMT.

8.4 Secondary variables

It cab be considered as secondary variables those that are part of humoral context discussed above, or that may result from disorders caused, as the onset of puberty (girls), the concentrations of adipokines and other factors that will be assessed.

The current knowledge of these factors and the role of adipose tissue as an endocrine organ are one of the most recent findings and many data are still provisional. However, this trial will take into account all contextual variables to help interpret the major changes and relationships among the factors that may influence this longitudinal study on the efficacy of metformin.

8.5 Description of the variables

⇒ Weight, height and body mass index (BMI): The body mass index is the starting point of the trial. All these parameters will be ascertained using routine methods with proven instruments (calibrated scale and Harpenden stadiometer). They will be expressed in absolute, percentile and standardized scores. These data will be necessary for the potential inclusion in the study (IMC ≥ p97 and <3 SD) for age, according to the tables mentioned above (16).

⇒ Weight and length at birth: In addition to the absolute values measured in gr. and cm (adjusted to the nearest 0.5 cm) with its decimals where appropriate, results will be expressed in percentiles according neonatal standard tables commonly used in clinics and adapted to the Spanish population (A. Ferrandez Longás et al. 16). Gestational age must be above 37 weeks and below 42 weeks. These values will be obtained from hospital medical records or relevant documents from primary care centers, and records of the collaborating centers.

⇒ Other biometric data: Such as body temperature (in Celsius degrees), the heart frequence, expressed in beats per minute, and blood pressure, in mmHg by the method of Riva-Rocci. To assess the degree of sexual maturation of the patients, a Tanner scale will be uses. All patients included in the trial must be in Tanner stage 1 at baseline. Other parameters of interest are obtained by means of diet and physical activity questionnaires.

⇒ Visceral fat area: visceral fat area will be measured as the area on magnetic resonance imaging (MRI) scan, as described (19). A multi-section 1.5 T system MRI device (Sigma LX General Electric) will be used with children placed on a suitable device without sedation. Axial T1 images through the abdomen and pelvis will be obtained using a 400 cm field of view and the following parameters: slice thickness 6 mm, repetition time 360 msec, time to echo 21 ms, 2 excitations, angle attack 90°, matrix 256x224, bandwidth 8.33. All images will be imported on the ADW 4.0 GE software.

The areas of subcutaneous and visceral adipose tissue will be measured by adapting an angled planimeter profile at the edges of the subcutaneous and visceral regions by a technician blind to data on birth weight and gestational age. Non-fat areas will be measured by the same procedure throughout the visceral area and the result will be subtracted from the total visceral region. The visceral fat area will be subdivided into intraperitoneal and retroperitoneal areas using the ascending and descending colon, the psoas profiles on each side of the spine and the highest point of the vessels above the vertebrae as planimeter references. The visceral area will be calculated by subtracting total areas of each organ of the peritoneal area. A group of measurements will be assessed by an independent technician and then perform a test of consistency with the rest of measurements. Also a cross section L3 level will be measured where measurements will also be taken.

The results of abdominal magnetic resonance will be expressed as:

Nuclear Magnetic Resonance (NMR)

Total area	mm2	
	Total fat	total fat
	Subcutaneous fat	sc fat
	Visceral fat	v fat

	Visc/sc fat ratio	v/sc
Level L3 section		
	Subcutaneous fat	sc fat
	Visceral fat	v fat

All these area numeric values can be treated statistically. We will consider the values that exceed the 90th centile.

MR imaging will allow the determination of intrahepatic lipid content (IHLC) which in adults is associated with insulin resistance and low adiponectin concentrations (31). Intrahepatic lipid content is calculated from regression equations comparing the densities of the liver, spleen and adipose tissue.

⇒ Body composition: A densitometric test using dual X-ray energy will be performed. A Lunar Prodigy (Lunar Corporation, Madison, USA) adapted to the appropriate software will be used, as described in (19). The device is adapted to pediatric measurements and emits a low radiation dose (0.1 milli Sievert in total). It measures bone mineral density and mineral content of the body, as well as the amount of lean mass and body fat, both in absolute (grams) and percentage values (%).

Body composition

BMD	BMC	FBM	FBM	TFM	TFM	AFM	AFM	LBM	LBM	STM	STFM
g/cm2	g	%	g	%	g	%	g	%	g	g	%

Acronims that correspond to the following parameters:

Initials	Parameters	Initials	Parameters
BMD	Bone Mineral Density	AFM	Abdominal Fat Mass
BMC	Bone Mineral Content	LBM	Lean Body Mass
FBM	Fat Body Mass	STM	Stimated Total Mass
TFM	Truncal Fat Mass	STFM	Stimated Tatal Fat Mass

All these parameters can be expressed in absolute values, as well as in standardized scores or centiles, in order to make appropriate comparisons.
The abdominal region will be defined as the region between the edge of the diaphragm and the intertrocanther line. All body composition parameters will be adjusted by height as recommended by Wells and Cole (32).

⇒ Hematology tests: Routine measurements of blood parameters will be performed in each patient. Of interest are possible alterations in leukocyte counts associated with low weight at birth (32). All data will be recorded on the case report form (CRF) as follows:

Parameter (acronym)	Units	Normal range
Erythrocytes (HEMT)	Mill/mmcc	4,00-5,20
Hemoglobin (HB)	g/dl	11,5-15,5

Hematocrit (HTO)	%	35,0-45,0
Leukocites (LEU)	Mil/mmcc	5,0-11,9
Lymphocytes (Lymph)	Mil/mmcc	1,5-5,0
Monocytes (Monos)	Mil/mmcc	0,1-0,7
Segmented (Segments)	Mil/mmcc	1,5-5,0
Eosinophils (Eos)	Mil/mmcc	0,0-0,5
Basophils (Bas)	Mil/mmcc	0,0-0,2
LUCA	Mil/mmcc	0,0-0,1
LUC%	%	0,0-0,5

⇒ Biochemistry tests: Routine biochemical tests will be performed during the trial:

Parameter (acronym)	Units	Normal range
Glucosa (GLUC)	mmol/L ♦ mg/dl	3,9-6,1 ♦ 70 - 109
Creatinine (CREA)	umol/L ♦ mg/dl	< 60,0 ♦ < 0,67
Alanin aminotransferase (ALT)	UI/L	2 - 31
Aspartate aminotransferase (AST)	UI/L	2 – 34

⇒ Insulin and insulin-like growth factor I (IGFI), in addition to glycemia, will be assessed in this clinical trial. The homeostatis model assessmet of insulin resistance and insulin secretion will be used as follows:

β Cell function= 20 [insulin (mU/L)] / [glucose (mmol/L) – 3,5]
Resist. Ins.(HOMA) = [insulin (mU/L)]x[glucose (mmol/L)]/ 22.5

This method proposed by Matthews et al. (46) allows estimating graphically the abovementioned parameters as follows:

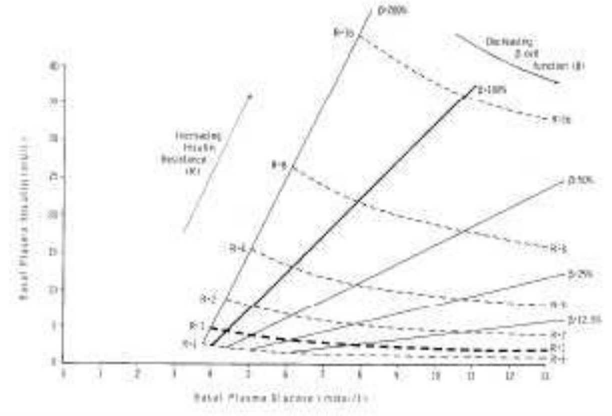

Y axis shows basal glucose levels, X axis shows basal insulin. Profiles obtained by the computer are drawn as a grid in which the continuous lines represent the percentage of β-cell function and the dotted lines represent the levels of insulin resistance for all subjects, the blood glucose concentrations being set by infusions (glycemic clamps). There is a computer program that estimates (HOMA-CIGMA Plan Calculator - Diabetes Research Laboratory - Oxford - UK)

these relationships (21) for each patient. Insulin values are determined by chemiluminescence with a sensitivity of 0.4 μ IU / mL.

For the IGF-I, a similar procedure to that for insulin will be used, with a sensitivity of 2.6 ng / mL. Increments in IGF-I are associated with insulin resistance during puberty.

⇒ Lipid profile: the following parameters will be analyzed:

Parameters (acronyms)	Units	Normal Range
Triglycerides (TRIG)	mmol/L	0,44-1,85
Total Cholesterol (COL TOT)	mmol/L	2,47-5,20
High Density Cholesterol (CHDL)	mmol/L	> 0,90
Low Densty Cholesterol (CLDL)	mmol/L	< 3,36

These parameters are part of the profile considered for the estimation of the cardiovascular risk.

⇒ Carotid intima-media thickness: It is considered a valuable parameter for assessing cardiovascular risk. It is measured by ultrasound according to a predefined protocol. The measurements are made on the common carotid with the patient in supine position after 10 minutes of rest and with the head slightly rotated ipsilateral to the measurement. The values of IMT (Intima-Media Thikness) are measured four times on each carotid and the mean values are used for the statistical analyses. Arterial intimal thickening is observed in obese people and in those with atherosclerotic disease.

⇒ Endocrine tests: We will perform the following endocrine tests:

Parameter (acronyms)	Units	Normal Range
Free tetraiodothyronine (FT4)	pmol/L	9,1 – 25,0
Thyrotropin (TSH)	mU/L	0,3 – 4,5
Insulin like growht factor I (IGFI)	ng/ml	60,00 – 380,00
Inmuno-reactive Insulin (IRI)	mU/L	< 19,5

The FT4 is the more useful parameter for thyroid function. It is measured by inmunochemiluminescence. It increases in hyperthyroidism and in cases of thyroid hormone resistance. It is assessed along with TSH levels, which allow explaining the relationship with the hypothalamus and pituitary gland. Sex hormones will be assessed in those patients in whom a pubertal onset is evidenced.

⇒Adipokines: page 37 at the beginning of this section describes the emerging role of adipokines, substances predominatly secreted by adipose tissue as regulators of multiple aspects of metabolism. Since the discovery of leptin and adiponectin in the mid 90's, new adipokines have been added to the group. For simplicity, the following table summarizes a selection of key adipokines and their effects:

Adipokine	Principal effects
Adiponectin	- Inflammation - Atherosclerosis - insulin resistance
Apelin	Insulin resistance, hemodynamic +, response to hypoxia
Chemerin	Involved in lipid and glucose metabolism
Plasminogen Activador Inhibidor-1	Atherogenic, insulin resistente
Interleukin-6	Type 2 diabetes mellitus and metabolic sindrome
Leptin	Anorectic, Reproduction, Angiogenesis, Inmunity, Atheriosclerosis
Omentin	Seems to regulate insulin action
Monocyte Chemokine Protein-1	T-lymphocyte recruitment
Resistin	Inflamation, Insulin Resistance, Pro-atherogenic
Tumor Necrosis Factor α	Insulin resistance, Atherogenic
Vaspin	Obesity, increases insulin sensitivity
Visfatin	Insulin resistance

Many of these adipokines exert not only endocrine, but also paracrine biological activities. Some contribute to maturation and sexual development, and therefore their role is far more complex. Throughout this trial, patterns that modulate the concentrations of adipokines will be studied. Adiponectin is considered a major adipokine in this trial.

⇒ Adiponectin: It is an important and abundant protein composed of 244 amino acids with structural similarity to collagen. It is present in the blood in large quantities, and accounts for up to 0.01% of total plasma proteins. Circulating adiponectin levels are proportional to the degree of obesity and insulin resistance, increasing with weight loss and the use of insulin sensitizing drugs. It is an adipokine that has been closely linked to metabolic syndrome. A number of beneficial or protective actions are known for this adipokine regulating. It is composed of lower- and high-molecular-weight forms and their levels are determined by ELISA and expressed as ng / mL.

Over the course of the trial, novel information may point to newer adipokines which will be ultimately assessed in stored sera.

8.6 Flowchart

The overall course and and assessments of the trial are shown in the following flowchart:

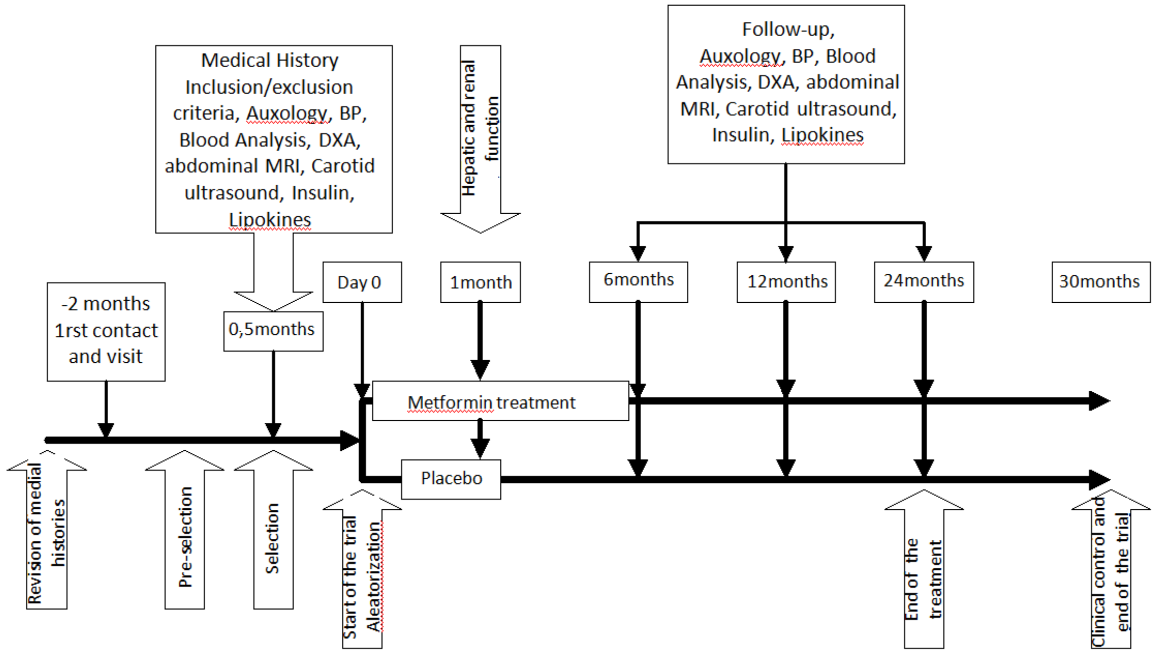

9. Adverse events

9.1. Definitions

Adverse event: it is any undesirable event occurring to a subject during a clinical trial, whether or not considered to be a consequence of the research product.

Serious adverse event: that which causes death, life threatening, permanent disability resulting in hospitalization or extension thereof.

Congenital anomalies and malignancies are always considered serious adverse effects.

Unexpected adverse events: it is an event not described (in nature, severity or frequency) in the investigator's brochure or in the product datasheet.

9.2 Information on adverse events occurring in a patient during the clinical trial

At each follow-up visits, the parents or guardians will be asked about the occurrence and nature of any adverse event. An adverse event is defined as any change in physiological or psychological state of the patient as compared to baseline data prior to the start of the study.

All adverse events will be reported by filling in the Case Report Form (CRF). The CRF for each adverse event shall contain the date of onset, severity and course over time.

Adverse events will be classified as mild, moderate or severe, as defined as follows:
⇒ Mild event: change in patient's clinical condition that does not affect its activity
⇒ Moderate event: change that causes slight alteration of their normal activity
⇒ Severe event: change that causes significant changes in their quality of life and usual activity.

9.3 Accountability criteria

All adverse events will be registered and assessed regardless of whether a causal relationship exists or not with the medication of the trial.

9.4 Procedures for immediate notification of serious or unexpected adverse events

The serious or unexpected adverse effects will be immediately notified to the study coordinator (Dr. Abel López Bermejo).

An adverse event is defined as serious in the following cases:

⇒ Threaten the patient's life
⇒ Requires Urgent Care
⇒ Requires hospitalization

9.5 Reporting of adverse events

An official form will be used for reporting adverse events, which is provided in **Appendix 3** of this protocol.

10. Ethical aspects

This trial will be conducted in compliance with all applicable legal and ethical requirements and standards of Good Clinical Practice ICH.

10.1. Clinical trial insurance

The initiation of a clinical trial with products in clinical investigation or new indications for drugs already approved or in the absence of therapeutic interest for the subject of the trial, would be only performed if there is an insurance to cover damages that the person might suffer as a consequence of the trial (Royal Decree 223/2004).

The trial sponsor is responsible for the recruitment of such liability insurance and this will cover the liability of the sponsor, the investigator and his staff and of the owners of the hospitals or centers where the trial is performed.

The minimum insured amount for civil liability will be 250,000 euros per subject participating in the clinical trial.

10.2. Subject Identification

The subject will be identified with a serial number and the number of trial in addition to the initials of the name and two surnames, in order to comply at all times with the rules of BPC and the confidentiality of information according to Law 15/1999. In the case report form, the process for using the census numbers are described; in section 5.2 the design and randomization processes are described.

10.3. Patient Information

The parents or guardians of patients will be informed of the non-invasive procedures for screening and selection, which will include the use of laboratory tests and additional examinations which may be considered minimally invasive. They will also be informed of the objectives and procedures of the trial and other details included in the standards of good clinical practice. Upon receiving all the information, they will be asked to sign the consent form. Patients aged between 6 and 10 years will be also verbally informed.

10.4. Consent form

Parents or guardians will sign a consent form if they wish to participate in the trial.

11. Practical Considerations

11.1 Special rules for patients and familiars

These rules are included in the patient information sheet. The clinical course differs little from the usual care given to patients with metabolic or endocrine abnormalities. At the end of the trial, the parents will be informed about the results and advised about the best care for their child.

11.2 Administrative issues

The protocol is presented to official entities for approval (Research Ethics Committee/IRB, Hospital Management, the Spanish Agency of Medicines and Health Products (AEMPS)).

11.2.1. Compliance and revisions of the protocol

The research coordinator will be responsible for compliance with all specifications of the protocol and convene meetings for supervising and reviewing the project according to specific procedures.

11.2.2. Trial supervision

The principal investigator and collaborators as well as the supervisor will work in accordance with the standards of BPC of ICH guidance. The sponsor will supervise the trial according to

specific procedures for ensuring quality of data and of the master file. If necessary, they will seek the services of an OCR, which will generate an amendment to the protocol.

11.2.3. Documents and reports

The trial documents: the master file (Trial Master File), as well as all progress reports / complementary or other reports that may have arisen according the particularities of the trial will be completed.

Upon completion of all the administrative stages and control of the drug under test, and the monitoring of all participating subjects, the clinical trial will be closed. A preliminary report will be elaborated and all the documents in the master file will be reviewed. All the trial documents will be kept according to the current legistation.

11.2.4. Relations with the Clinical Research Ethics Comite

All aspects of the trial will be approved by the Committee of Reference. Any modification or amendment to a previously approved protocol must be reported to the CEIC/IRB and all relevant amendments must be approved before they can be implemented.

11.2.5. Policy of publications

The results obtained in the clinical trial will be reviewed and discussed by the research team and the sponsor for publication.

The data obtained will not be disseminated to third parties until a consensus is reached for its disclosure, either in the form of a conference, communication or publication, taking also into account the current legislation of the Ministry of Health. Researchers may include the assay title in their abstract. The title of the assay will appear in the annual report of participating services and hospitals.

11.3 Product under research

The product under research is the drug marketed by Kern Pharma SL as metformin tablets 850 mg. that requires no special features of conservation if temperature does not exceed 25 º C. The placebo does not require special specifications. The Pharmacy will maintain both products under controlled conditions according to common protocols used for Clinical Trial.

11.3.1. Product identification

The product must be properly labeled to ensure the traceability of the study, according to quality system standards of the Hospital Pharmacy Department as recorded in its regulations for dispensing products for clinical trials. Both patients and researchers can not distinguish the experimental product from the placebo.

11.3.2. Management and delivery

The principal investigator will have the necessary daily amounts of the test drug and will keep under adequate conditions for use as directed by the Hospital Pharmacy Service.

11.3.3. Packaging and labeling

All study medication will be packaged and labeled according to specific protocol. The Pharmacy will keep a record of the identification of the lots and products necessary for tracing.

11.3.4. Supply applications

The request of the drug for the trial will be performed by the principal investigator at the Pharmacy, using the request document included in the section of documents and related records.

11.3.5. Deposit of the research product

The product under investigation will be kept in the Pharmacy and delivered according to the specific protocols for dispensing products for clinical trial.

11.3.6. Return of the research product

Upon completion of the trial or of the phase of drug use, the principal investigator of the medication will revise the trial and register all the medication used in the trial and all of the remaining medication. Then, the principal investigator will return all of the remaining medication to the Pharmacy service, using a prespecified procedure.

11.3.7. Destruction of the research product

The Pharmacy Service will return all of the remaining trial medication to the trial sponsor who will acknowledge its receipt by signing a document, and will follow the procedures that are prespecified for its destruction.

12. Statistical Analysis

12.1 General plan

The statistical analysis focuses on a global quantitative evaluation and correct interpretation of test results to make a conclusion on the efficacy or not of the preparation used and the safety conditions used in the trial.

Includes the following:

Case Study:

Clinical and safety data

Additional examinations
Evaluation of primary and secondary endpoints

12.2 Descriptive statistics and data evaluation

The variables (categorical and quantitative) will be collected initially in an Excel database. The data will be downloaded to a SPSS (version 12.0) dataset and used for statistical analysis. This will include statistical tests to determine the distribution of the data (Kolmogorov-Smirnov) to compare and correlate quantitative data. Because of the limited number of cases, statistical tests for non-Gaussian data distributions, such as the Mann-Whitney, will be used to compare two independent samples. Wilcoxon test for paired data will be used for follow-up data.

Parametric tests to be applied will be the Student t test and ANOVA with Bonferroni correction to compare various groups. Linear regression analysis will be used for correlations of more than two variables (one dependent and two or more explanatory variables). Logistic regression models will be used for variables whose relationship does not follow linear functions. Pearson Chi-square will be used to look for associations between categorical variables.

13. References

1. Ogden CL, Yanovski SZ, Carroll MD, Flegal KM. The epidemiology of obesity. Gastroenterology 2007;132:2087-102.
2. Serra-Majem L, Aranceta Bartrina J, Perez-Rodrigo C, Ribas-Barba L, Delgado-Rubio A. Prevalence and deteminants of obesity in Spanish children and young people. Br J Nutr 2006;96 Suppl 1:S67-72.
3. Effect of intensive blood-glucose control with metformin on complications in overweight patients with type 2 diabetes (UKPDS 34). UK Prospective Diabetes Study (UKPDS) Group. Lancet 1998;352:854-65.
4. Knowler WC, Barrett-Connor E, Fowler SE, Hamman RF, Lachin JM, Walker EA, et al. Reduction in the incidence of type 2 diabetes with lifestyle intervention or metformin. N Engl J Med 2002;346:393-403.
5. Freemark M, Bursey D. The effects of metformin on body mass index and glucose tolerance in obese adolescents with fasting hyperinsulinemia and a family history of type 2 diabetes. Pediatrics 2001;107:E55.
6. Park MH, Kinra S, Ward KJ, White B, Viner RM. Metformin for obesity in children and adolescents: a systematic review. Diabetes Care 2009;32:1743-5.
7. Ibanez L, Lopez-Bermejo A, Diaz M, Marcos MV, de Zegher F. Metformin treatment for four years to reduce total and visceral fat in low birth weight girls with precocious pubarche. J Clin Endocrinol Metab 2008;93:1841-5.
8. Ibanez L, Ong K, Valls C, Marcos MV, Dunger DB, de Zegher F. Metformin treatment to prevent early puberty in girls with precocious pubarche. J Clin Endocrinol Metab 2006;91:2888-91.
9. Ibanez L, Lopez-Bermejo A, Diaz M, Marcos MV, de Zegher F. Early metformin therapy to delay menarche and augment height in girls with precocious pubarche. Fertil Steril 2010;(en prensa).
10. Dolinoy DC, Jirtle RL. Environmental epigenomics in human health and disease. Environ Mol Mutagen 2008;49:4-8.

11. Ibanez L, Lopez-Bermejo A, Diaz M, Marcos MV, de Zegher F. Pubertal metformin therapy to reduce total, visceral, and hepatic adiposity. J Pediatr 2010;156:98-102 e1.
12. Ibanez L, Valls C, Marcos MV, Ong K, Dunger DB, De Zegher F. Insulin sensitization for girls with precocious pubarche and with risk for polycystic ovary syndrome: effects of prepubertal initiation and postpubertal discontinuation of metformin treatment. J Clin Endocrinol Metab 2004;89:4331-7.
13. Burke GL, Bertoni AG, Shea S, Tracy R, Watson KE, Blumenthal RS, et al. The impact of obesity on cardiovascular disease risk factors and subclinical vascular disease: the Multi-Ethnic Study of Atherosclerosis. Arch Intern Med 2008;168:928-35.
14. Zhou G, Myers R, Li Y, Chen Y, Shen X, Fenyk-Melody J, et al. Role of AMP-activated protein kinase in mechanism of metformin action. J Clin Invest 2001;108:1167-74.
15. Schwimmer JB, et al. A phase 2 clinical trial of metformin as a treatment for non-diabetic paediatric non-alcoholic steatohepatitis. Aliment Pharmacol Ther 2005; 21:871-9.
16. Ferrández-Longás A, et al. Estudio longitudinal de crecimiento y desarrollo. Centro Andrea Prader. Zaragoza 1980-2002. In ERGON, ed. Patrones de crecimiento y desarrollo en España., pp 61-115. Madrid: 2004.
17. Marshall WA, Tanner JM. Variations in pattern of pubertal changes in girls. Arch Dis Child 1969; 44:291-303.
18. Marshall WA, Tanner JM. Variations in pattern of pubertal changes in boys. Arch Dis Child 1970; 45:13-23.
19. Ibáñez L, et al. Early development of visceral fat excess following spontaneous catch-up growth in children with low birthweight. J Clin Endocrinol Metab 2008; 93:925-8.
20. Ibáñez L, et al. Insulin sensitization early post-menarche prevents progression from precocious pubarche to polycystic ovary syndrome. J Pediatr 2004; 144:23-9.
21. Ibáñez L, et al. Metformin treatment for four years to reduce total and visceral fat in low birth weight girls with precocious pubarche. J Clin Endocrinol Metab 2008 ; 93:1841-5.
22. Ibáñez L, et al. Pubertal adiposity after fetal growth restraint: toward a calorie restriction mimetic approach. Metabolism 2008; 57:672-5.
23. Stumvoll M, et al. Metformin. Endocr Res 2007; 32:39-57.
24. Kanazawa I, Yamaguchi T, Yano S, Yamauchi M, Sugimoto T. Metformin enhances the differentiation and mineralization of osteoblastic MC3T3-E1 cells via AMP kinase activation as well as eNOS and BMP-2 expression. Biochem Biophys Res Commun 2008; 375:414-419.
25. Ibáñez L, et al. Precocious pubarche, hyperinsulinism and ovarian hyperandrogenism in girls: relation to reduced fetal growth. J Clin Endocrinol Metab 1998; 83:3558-662.
26. Antuna-Puente B, Feve B, Fellahi S, Bastard JP. Adipokines: the missing link between insulin resistance and obesity. Diabetes Metab 2008; 34:2-11.
27. Campos DB, Palin MF, Bordignon V, Murphy BD. The 'beneficial' adipokines in reproduction and fertility. Int J Obes (Lond) 2008; 32:223-231.
28. MacLaren R, Cui W, Cianflone K. Adipokines and the immune system: an adipocentric view. Adv Exp Med Biol 2008; 632:1-21.
29. Greulich WW, Pyle SI. Radiographic atlas of skeletal development of the hand and wrist. Standford: Stanford University Press; 1959.
30. Koska J, et al. Increased fat accumulation in liver may link insulin resistance with subcutaneous abdominal adipocyte enlargement, visceral adiposity, and hypoadiponectinemia in obese individuals. Am J Clin Nutr 2008; 87:295-302.

31. Koska J, et al. Increased fat accumulation in liver may link insulin resistance with subcutaneous abdominal adipocyte enlargement, visceral adiposity, and hypoadiponectinemia in obese individuals. Am J Clin Nutr 2008; 87:295-302.
32. Wells JC, Cole TJ. Adjustment of fat-free mass and fat mass for height in children aged 8 y. Int J Obes Relat Metab Disord 2002; 26: 947-952.

Apendix 1

Acronyms gloosario:

Siglas	Significado
AEMPS	Spanish Agency of Medicines and Medical Devices
BMI	Body Mass Index
DHEAS	DeHydroEpiAndrosterone Sulfate
HDL	High Density Lipoproteins
HMW	High Molecular Weight
HOMA	Homeostatic Model Assessment
IMT	Intima Media Thikness
LDL	Low Density Lipoproteins
MSI	Mean Serum Insulin
MS	Metabolic Syndrome
PP	Precocious pubarche
RM	Magnetic Resonance
SHBG	Sex Hormone Binding Globulin

Apendix 2

Declaración of Helsinki (Updated 2008)

Adopted by the 18th WMA General Assembly, Helsinki, Finland, June 1964, and amended by:
29th WMA General Assembly, Tokyo, Japan, October 1975
35th WMA General Assembly, Venice, Italy, October 1983
41st WMA General Assembly, Hong Kong, September 1989
48th WMA General Assembly, Somerset West, Republic of South Africa, October 1996
52nd WMA General Assembly, Edinburgh, Scotland, October 2000
53rd WMA General Assembly, Washington 2002 (Note of Clarification on paragraph 29 added)
55th WMA General Assembly, Tokyo 2004 (Note of Clarification on Paragraph 30 added)
59th WMA General Assembly, Seoul, October 2008

A. INTRODUCTION

1. The World Medical Association (WMA) has developed the Declaration of Helsinki as a statement of ethical principles for medical research involving human subjects, including research on identifiable human material and data.
   The Declaration is intended to be read as a whole and each of its constituent paragraphs should not be applied without consideration of all other relevant paragraphs.

2. Although the Declaration is addressed primarily to physicians, the WMA encourages other participants in medical research involving human subjects to adopt these principles.

3. It is the duty of the physician to promote and safeguard the health of patients, including those who are involved in medical research. The physician's knowledge and conscience are dedicated to the fulfilment of this duty.

4. The Declaration of Geneva of the WMA binds the physician with the words, "The health of my patient will be my first consideration," and the International Code of Medical Ethics declares that, "A physician shall act in the patient's best interest when providing medical care."

5. Medical progress is based on research that ultimately must include studies involving human subjects. Populations that are underrepresented in medical research should be provided appropriate access to participation in research.

6. In medical research involving human subjects, the well-being of the individual research subject must take precedence over all other interests.

7. The primary purpose of medical research involving human subjects is to understand the causes, development and effects of diseases and improve preventive, diagnostic and therapeutic interventions (methods, procedures and treatments). Even the best current interventions must be evaluated continually through research for their safety, effectiveness, efficiency, accessibility and quality.

8. In medical practice and in medical research, most interventions involve risks and burdens.

9. Medical research is subject to ethical standards that promote respect for all human subjects and protect their health and rights. Some research populations are particularly vulnerable and need special protection. These include those who cannot give or refuse consent for themselves and those who may be vulnerable to coercion or undue influence.

10. Physicians should consider the ethical, legal and regulatory norms and standards for research involving human subjects in their own countries as well as applicable international norms and standards. No national or international ethical, legal or regulatory requirement should reduce or eliminate any of the protections for research subjects set forth in this Declaration.

B. BASIC PRINCIPLES FOR ALL MEDICAL RESEARCH

11. It is the duty of physicians who participate in medical research to protect the life, health, dignity, integrity, right to self-determination, privacy, and confidentiality of personal information of research subjects.

12. Medical research involving human subjects must conform to generally accepted scientific principles, be based on a thorough knowledge of the scientific literature, other relevant sources of information, and adequate laboratory and, as appropriate, animal experimentation. The welfare of animals used for research must be respected.

13. Appropriate caution must be exercised in the conduct of medical research that may harm the environment.

14. The design and performance of each research study involving human subjects must be clearly described in a research protocol. The protocol should contain a statement of the ethical considerations involved and should indicate how the principles in this Declaration have been addressed. The protocol should include information regarding funding, sponsors, institutional affiliations, other potential conflicts of interest, incentives for subjects and provisions for treating and/or compensating subjects who are harmed as a consequence of participation in the research study. The protocol should describe arrangements for post-study access by study subjects to interventions identified as beneficial in the study or access to other appropriate care or benefits.

15. The research protocol must be submitted for consideration, comment, guidance and approval to a research ethics committee before the study begins. This committee must be independent of the researcher, the sponsor and any other undue influence. It must take into consideration the laws and regulations of the country or countries in which the research is to be performed as well as applicable international norms and standards but these must not be allowed to reduce or eliminate any of the protections for research subjects set forth in this Declaration. The committee must have the right to monitor ongoing studies. The researcher must provide monitoring information to the committee, especially information about any serious adverse events. No change to the protocol may be made without consideration and approval by the committee.

16. Medical research involving human subjects must be conducted only by individuals with the appropriate scientific training and qualifications. Research on patients or healthy volunteers

requires the supervision of a competent and appropriately qualified physician or other health care professional. The responsibility for the protection of research subjects must always rest with the physician or other health care professional and never the research subjects, even though they have given consent.

17. Medical research involving a disadvantaged or vulnerable population or community is only justified if the research is responsive to the health needs and priorities of this population or community and if there is a reasonable likelihood that this population or community stands to benefit from the results of the research.

18. Every medical research study involving human subjects must be preceded by careful assessment of predictable risks and burdens to the individuals and communities involved in the research in comparison with foreseeable benefits to them and to other individuals or communities affected by the condition under investigation.

19. Every clinical trial must be registered in a publicly accessible database before recruitment of the first subject.

20. Physicians may not participate in a research study involving human subjects unless they are confident that the risks involved have been adequately assessed and can be satisfactorily managed. Physicians must immediately stop a study when the risks are found to outweigh the potential benefits or when there is conclusive proof of positive and beneficial results.

21. Medical research involving human subjects may only be conducted if the importance of the objective outweighs the inherent risks and burdens to the research subjects.

22. Participation by competent individuals as subjects in medical research must be voluntary. Although it may be appropriate to consult family members or community leaders, no competent individual may be enrolled in a research study unless he or she freely agrees.

23. Every precaution must be taken to protect the privacy of research subjects and the confidentiality of their personal information and to minimize the impact of the study on their physical, mental and social integrity.

24. In medical research involving competent human subjects, each potential subject must be adequately informed of the aims, methods, sources of funding, any possible conflicts of interest, institutional affiliations of the researcher, the anticipated benefits and potential risks of the study and the discomfort it may entail, and any other relevant aspects of the study. The potential subject must be informed of the right to refuse to participate in the study or to withdraw consent to participate at any time without reprisal. Special attention should be given to the specific information needs of individual potential subjects as well as to the methods used to deliver the information. After ensuring that the potential subject has understood the information, the physician or another appropriately qualified individual must then seek the potential subject's freely-given informed consent, preferably in writing. If the consent cannot be expressed in writing, the non-written consent must be formally documented and witnessed.

25. For medical research using identifiable human material or data, physicians must normally seek consent for the collection, analysis, storage and/or reuse. There may be situations where

consent would be impossible or impractical to obtain for such research or would pose a threat to the validity of the research. In such situations the research may be done only after consideration and approval of a research ethics committee.

26. When seeking informed consent for participation in a research study the physician should be particularly cautious if the potential subject is in a dependent relationship with the physician or may consent under duress. In such situations the informed consent should be sought by an appropriately qualified individual who is completely independent of this relationship.

27. For a potential research subject who is incompetent, the physician must seek informed consent from the legally authorized representative. These individuals must not be included in a research study that has no likelihood of benefit for them unless it is intended to promote the health of the population represented by the potential subject, the research cannot instead be performed with competent persons, and the research entails only minimal risk and minimal burden.

28. When a potential research subject who is deemed incompetent is able to give assent to decisions about participation in research, the physician must seek that assent in addition to the consent of the legally authorized representative. The potential subject's dissent should be respected.

29. Research involving subjects who are physically or mentally incapable of giving consent, for example, unconscious patients, may be done only if the physical or mental condition that prevents giving informed consent is a necessary characteristic of the research population. In such circumstances the physician should seek informed consent from the legally authorized representative. If no such representative is available and if the research cannot be delayed, the study may proceed without informed consent provided that the specific reasons for involving subjects with a condition that renders them unable to give informed consent have been stated in the research protocol and the study has been approved by a research ethics committee. Consent to remain in the research should be obtained as soon as possible from the subject or a legally authorized representative.

30. Authors, editors and publishers all have ethical obligations with regard to the publication of the results of research. Authors have a duty to make publicly available the results of their research on human subjects and are accountable for the completeness and accuracy of their reports. They should adhere to accepted guidelines for ethical reporting. Negative and inconclusive as well as positive results should be published or otherwise made publicly available. Sources of funding, institutional affiliations and conflicts of interest should be declared in the publication. Reports of research not in accordance with the principles of this Declaration should not be accepted for publication.

C. ADDITIONAL PRINCIPLES FOR MEDICAL RESEARCH COMBINED WITH MEDICAL CARE

31. The physician may combine medical research with medical care only to the extent that the research is justified by its potential preventive, diagnostic or therapeutic value and if the physician has good reason to believe that participation in the research study will not adversely affect the health of the patients who serve as research subjects.

32. The benefits, risks, burdens and effectiveness of a new intervention must be tested against those of the best current proven intervention, except in the following circumstances:

- The use of placebo, or no treatment, is acceptable in studies where no current proven intervention exists; or
- Where for compelling and scientifically sound methodological reasons the use of placebo is necessary to determine the efficacy or safety of an intervention and the patients who receive placebo or no treatment will not be subject to any risk of serious or irreversible harm. Extreme care must be taken to avoid abuse of this option.

33. At the conclusion of the study, patients entered into the study are entitled to be informed about the outcome of the study and to share any benefits that result from it, for example, access to interventions identified as beneficial in the study or to other appropriate care or benefits.

34. The physician must fully inform the patient which aspects of the care are related to the research. The refusal of a patient to participate in a study or the patient's decision to withdraw from the study must never interfere with the patient-physician relationship.

35. In the treatment of a patient, where proven interventions do not exist or have been ineffective, the physician, after seeking expert advice, with informed consent from the patient or a legally authorized representative, may use an unproven intervention if in the physician's judgement it offers hope of saving life, re-establishing health or alleviating suffering. Where possible, this intervention should be made the object of research, designed to evaluate its safety and efficacy. In all cases, new information should be recorded and, where appropriate, made publicly available.

Apendix 3 – Form of adverse event reporting

**NOTIFICATION OF ADVERSE EVENTS**<br>PRODUCT IN CLINICAL RESEARCH PAHSE	PROTOCOL Nº	NOTIFICACION Nº (Laboratory)
	PATIENT Nº	NOTIFICATION Nº

I. INFORMATION ON THE ADVERSE EVENT

1.PATIENT INITIALS	1.a. COUNTRY	2. BIRTH DATE	2a.AGE	3. SEX	3ª.WEIGHT	4-6. START OF rEACTION			8. CONSEQUENCES
						DAY	MONTH	YEAR	☐ DEATH<br>☐ PATIENT'S LIFE HAS BEEN IN DANGER<br>☐ HOSPITALIZTION OR PROLONGATION OF HOSPTITALIZATION<br>☐ PERMANENT OR SIGNIFICANT DISABILITY<br>☐ PERSISTENCE OF ADVERSE EVENT<br>☐ RECOVERY
7. DESCRIPTION OF ADVERSE EVENT (Including relevant exploratory or laboratory results)									

II. PRODUCT INFORMATION RESEARCH

14. NAME		20. ¿ADVERSE EVENT PASSES AFTER STOP THE MEDICATION?<br>☐ Yes ☐ No ☐ Not applicable
15. DAILY DOSE	16. ROUTE OF ADMINISTRATION	21. ¿REAPPEARS TEH ADVERSE EVENT AFTER GIVING BACH THE MEDICATION?<br>☐ Yes ☐ No ☐ Not applicable
17. DISEASE STUDY		
18. DATES OF TREATMETN (From / To)	19. TREATMENT DURATION	

III. MEDICAL HISTORY AND CONCOMITANT DRUG

22. CONCOMITANT DRUGS AND DATE OF ADMINISTRATION
23. IMPORTANT INFORMATION OF THE CLINIC HISTORY (ej. diagnoses, allergies, pregnancy, etc)

IV. INFORMATION ABOUT THE SPONSOR AND THE INVESTIGADOR

24a. NAME AND ADDRESS OF THE TRIAL SPONSOR		24b. NAME AND ADDRESS OF INVESTIGATOR
24d. LABORATORY CODE BY DGFPS	25a. TYPE OF REPORT<br>☐ INITIAL<br>☐ MONITORING	24c. SPONSOR'S TECHNITIAN WHO INFORMS<br>NAME:<br>TELEPHONE:<br>SIGNATURE:
24e. REPORT DATE	25b. DGFPS DATE	25c. ☐ SUPPLEMENTARY REPORT ATTACHED

Apendix 4

Neonatal Standard tables

Male

Lenght (cms) expressed as a mean ± SD

Edat Gestacional	Longitud	DE
**26**	34.39	1.96
**27**	35.98	1.77
**28**	37.26	1.65
**29**	37.99	1.43
**30**	39.68	1.79
**31**	40.85	1.38
**32**	42.22	1.69
**33**	43.25	1.61
**34**	44.93	1.77
**35**	45.98	1.81
**36**	47.36	1.87
**37**	48.50	1.89
**38**	49.47	1.68
**39**	49.99	1.68
**40**	50.38	1.66
**41**	50.78	1.72
**42**	51.46	1.84

Weight (grams) expressed as a mean ± SD

Edat Gestacional	Pes	DE
**26**	844.17	130.68
**27**	969.59	163.38
**28**	1097.19	207.50
**29**	1204.91	180.34
**30**	1394.38	210.67
**31**	1562.83	223.70
**32**	1749.29	283.28
**33**	1940.00	270.22
**34**	2201.24	298.03
**35**	2421.09	341.31
**36**	2639.74	351.11
**37**	2904.48	442.35
**38**	3149.31	405.14
**39**	3300.41	396.85
**40**	3398.72	398.39
**41**	3480.59	401.25
**42**	3617.89	435.67

Women

Lenght (cms) expressed as a mean ± SD

Edat Gestacional	Longitud	DE
**26**	34.06	1.81
**27**	35.37	1.63
**28**	37.00	1.34
**29**	37.91	1.06
**30**	39.74	1.91
**31**	40.93	2.21
**32**	41.75	1.39
**33**	42.99	1.92
**34**	44.26	1.78
**35**	45.58	1.79
**36**	46.57	2.07
**37**	47.86	1.98
**38**	48.68	1.67
**39**	49.43	1.66
**40**	49.81	1.66
**41**	50.11	1.59
**42**	50.58	1.53

Weight (grams) expressed as a mean ± SD

Edat Gestacional	Pes	DE
**26**	789.00	104.44
**27**	918.02	128.56
**28**	1041.56	176.29
**29**	1231.76	268.34
**30**	1347.13	254.30
**31**	1547.82	290.78
**32**	1638.26	318.23
**33**	1900.03	297.77
**34**	2159.60	307.44
**35**	2310.42	333.57
**36**	2522.76	393.15
**37**	2811.10	417.42
**38**	2982.84	376.71
**39**	3185.00	372.08
**40**	3279.90	372.30
**41**	3349.45	401.54
**42**	3469.80	383.54

Apendix 5: Growth charts

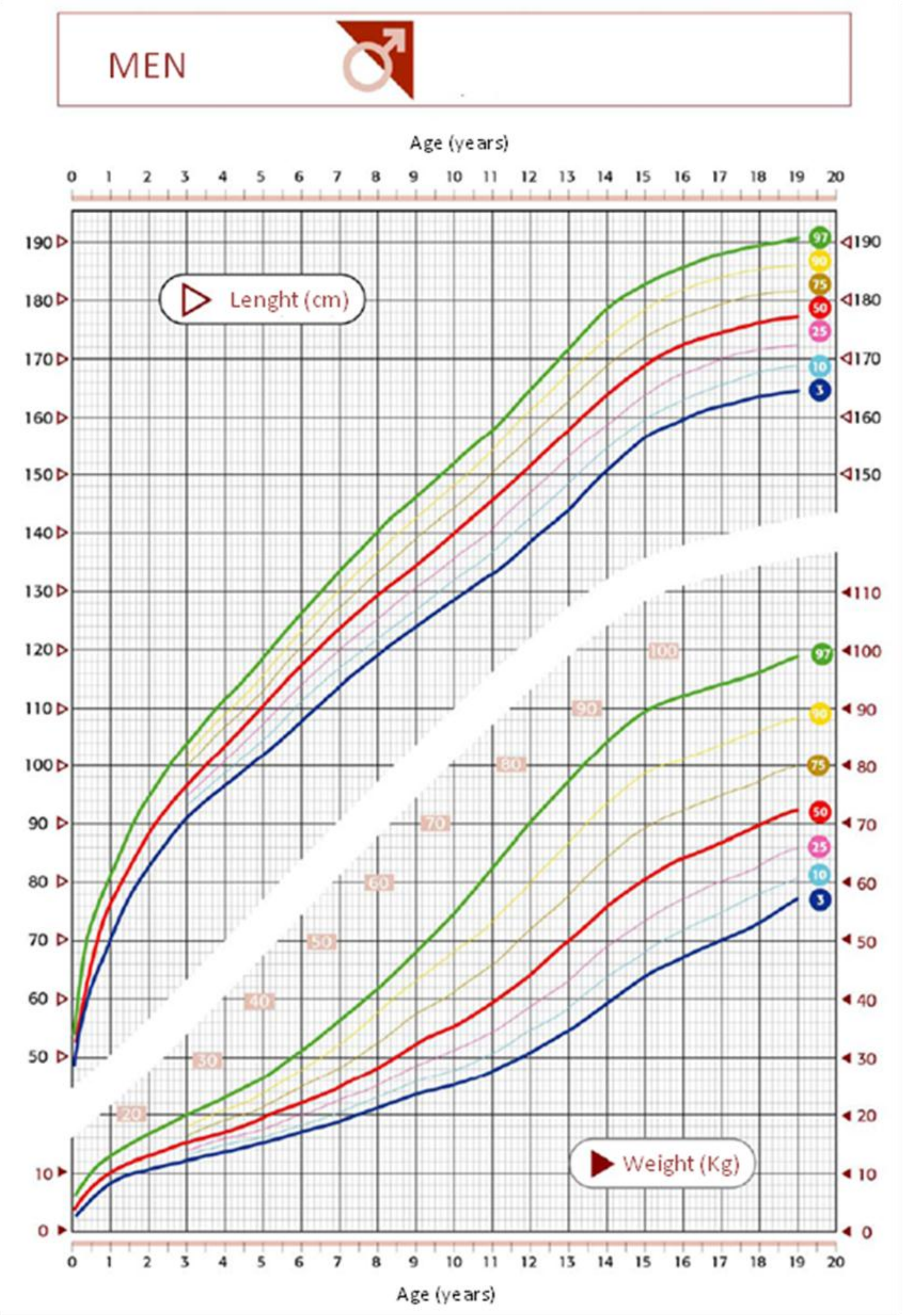

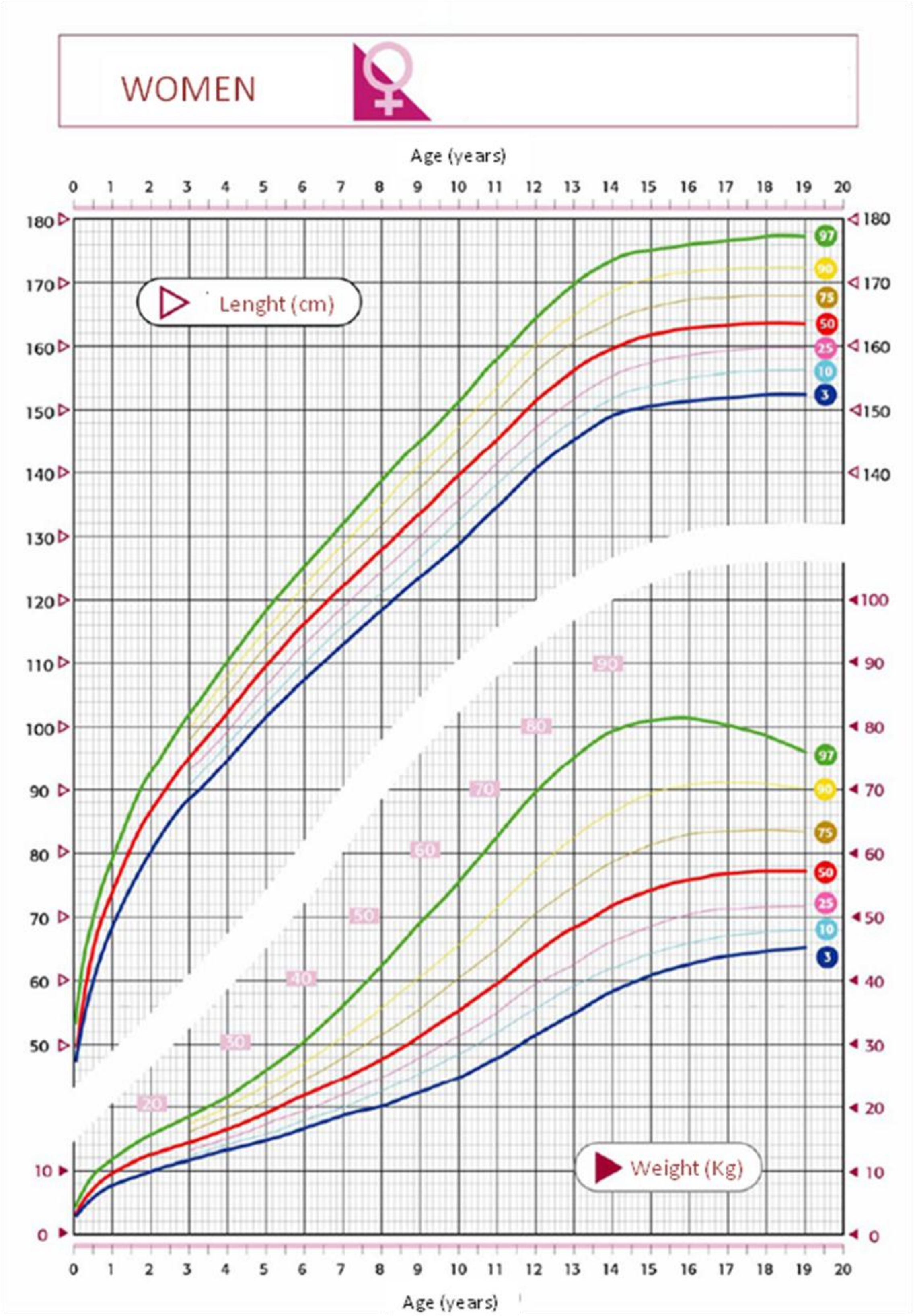

WOMEN
Age (years)
Lenght (cm)
Weight (Kg)
0 1 2 3 4 5 6 7 8 9 10 11 12 13 14 15 16 17 18 19 20
180 170 160 150 140 130 120 110 100 90 80 70 60 50 10 0
100 90 80 70 60 50 40 30 20 10 0
97 90 75 50 25 10 3
